# Single-cell transcriptomics reveals bladder microenvironment dynamics in Hunner type interstitial cystitis

**DOI:** 10.1016/j.isci.2026.116725

**Published:** 2026-07-09

**Authors:** Fumihiko Urabe, Kentaro Yoshihara, Jun Nakayama, Yuta Hirano, Naoaki Watanabe, Hironori Suzuki, Miyaka Umemori, Kagenori Ito, Taro Igarashi, Shun Sato, Takahiro Kimura, Akira Furuta, Yusuke Yamamoto

**Affiliations:** 1 Department of Urology, The Jikei University School of Medicine, Tokyo, Japan; 2 Laboratory of Integrative Oncology, National Cancer Center Research Institute, Tokyo, Japan; 3 Division of Cell Signaling and Informatics, Cancer Research Institute, Kanazawa University, Kanazawa, Ishikawa, Japan; 4 Bioinformatics and Omics Unit, Collaborative Research Promotion Domain, Next-Generation Precision Cancer Research Center, Osaka International Cancer Institute, Osaka, Japan; 5 Division of Respiratory Diseases, Department of Internal Medicine, The Jikei University School of Medicine, Tokyo, Japan; 6 Department of Clinical Laboratory Technology, Faculty of Medical Science, Juntendo University, Chiba, Japan; 7 Department of Pathology, The Jikei University School of Medicine, Tokyo, Japan

**Keywords:** interstitial cystitis, single-cell RNA-seq, fibroblast epithelial communication

## Abstract

Hunner type interstitial cystitis is a chronic inflammatory bladder disorder characterized by pain and severe lower urinary tract symptoms. Using single-cell RNA sequencing of Hunner lesions, non-Hunner lesions, and control bladder tissues, we characterized cellular states and intercellular communication within the bladder microenvironment. We identified a neuregulin enriched fibroblast population that was preferentially expanded in Hunner lesions and exhibited enhanced signaling toward basal epithelial cells through NRG and WNT pathways. In contrast, myofibroblast states were more prominent in non-Hunner lesions. Epithelial cells showed increased heterogeneity and differentiation toward inflammatory umbrella cell states, consistent with tissue remodeling. These findings define stromal and epithelial alterations across the diseased bladder and highlight fibroblast epithelial communication as a potential contributor to chronic inflammation and tissue remodeling in Hunner type interstitial cystitis.

## Highlights

• Single-cell transcriptomics maps cellular states in Hunner type IC
• NRG enriched fibroblasts are expanded in Hunner lesions
• Fibroblast epithelial signaling is enhanced through NRG and WNT pathways
• Epithelial and immune heterogeneity increases with disease progression

## Graphical abstract

**Figure:**
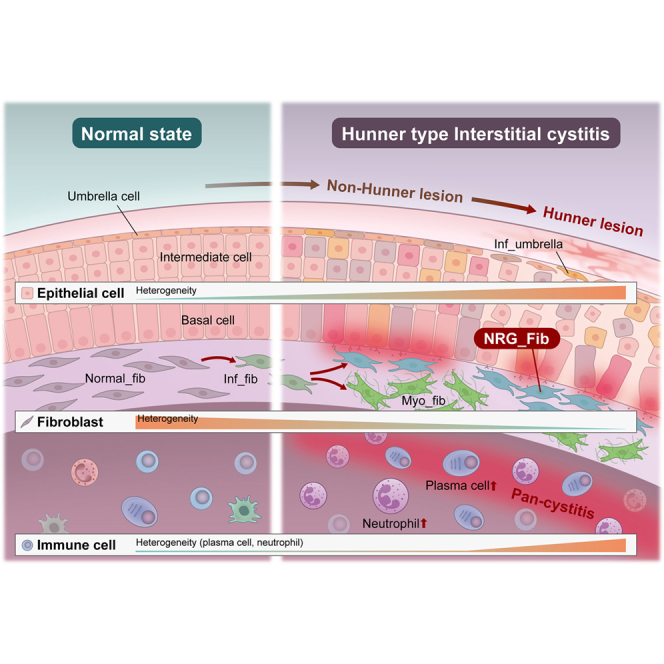

## Teaser

Immunology; Pathology; Genomics; Transcriptomics

## Introduction

Interstitial cystitis/bladder pain syndrome (IC/BPS) is a chronic debilitating disorder characterized by persistent bladder/urethral pain and lower urinary tract symptoms such as urinary frequency and urgency.^1^ IC/BPS represents a symptomatic syndrome complex that can be classified into two distinct entities, depending on the presence or absence of Hunner lesions.^2^ IC/BPS accompanied by Hunner lesions, hereafter referred to as Hunner-type IC (HIC) according to the East Asian guidelines for IC/BPS, whereas IC/BPS without Hunner lesions is classified as bladder pain syndrome (BPS). IC/BPS lacking Hunner lesions, hereafter referred to as BPS.^1^

Histologically, HIC is characterized by epithelial denudation and chronic inflammatory changes including stromal edema, neovascularization, and dense inflammatory infiltrates composed predominantly of lymphoplasmacytic and mast cells.^3^^,^^4^^,^^5^^,^^6^ Of note, these inflammatory changes and epithelial denudation can be observed not only in Hunner lesions, but also in the whole bladder wall including non-lesion bladder mucosa, indicating that HIC is a pancystitic disease.^4^^,^^6^ In a previous RNA-seq analysis, Akiyama et al. identified a set of biological pathways that are significantly enriched in HIC relative to BPS and normal bladders. However, in that study, no significant altered biological pathways between the Hunner lesion and non-lesion area of HIC was detected.^7^ The similar genomic and histological characteristics of Hunner lesion and non-lesion areas are consistent with the pancystitic nature of HIC. However, the distinctive reddish appearance of Hunner lesions and their predilection for particular bladder sites prompted us to perform a more in-depth analysis.

Single-cell RNA sequencing (scRNA-seq) is a powerful tool for investigating intra-disease heterogeneity and analyzing the gene expression profiles of rare cell populations. To date, two studies have utilized scRNA-seq to examine HIC, both of which primarily focused on immune cells.^8^^,^^9^ In our study, we collected samples from Hunner lesions, non-Hunner lesions, and non-inflamed control bladder tissues (normal bladder tissue) to perform scRNA-seq. Our aim was to explore not only immune cells but also other cell types that contribute to the pathology of interstitial cystitis and to investigate the intercellular communication occurring in the disease context.

## Results

### Single-cell transcriptome atlas of Huner lesion, non-Hunner lesions, and non-cystitis controls

**Figure 1:**
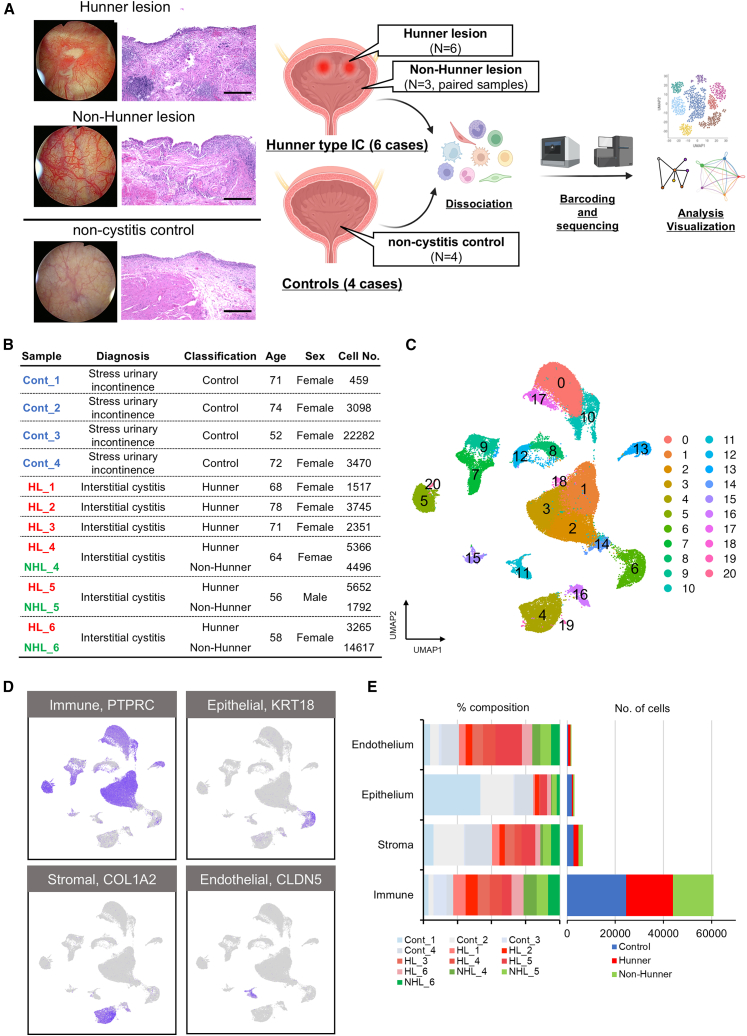
Mapping the cellular landscape of Hunner lesion, non-Hunner lesions, and non-cystitis controls by single-cell RNA sequencing (scRNA-seq) (A) Experimental workflow of scRNA-seq analysis. A total of 6 Hunner lesion samples, 3 non-Hunner lesion samples, and 4 non-cystitis controls were analyzed. (scale bar: 300 μm). (B) Summary table showing patient information, including ID, diagnosis, age, sex, and the number of cells captured for each sample. Cont indicates controls, HL indicates Hunner lesions, and NHL indicates non-Hunner lesions. (C) UMAP plot displaying the major cell clusters identified across all bladder samples (total cell number: 72,110). (D) UMAP plots showing the expression of representative marker genes: *PTPRC* for immune cells, *KRT18* for epithelial cells, *COL1A2* for stromal cells, and *CLDN5* for endothelial cells. (E) Proportional distribution of each major cell type (*x* axis) based on the UMAP plot in Figure 1D. Subsequently, the number of endothelial, epithelial, and stromal cells were calculated.

We performed single-cell transcriptome profiling on 6 Hunner lesion, 3 non-Hunner lesion, and 4 non-cystitis control samples (Figure 1A). Normal bladder tissues were obtained from a biopsy specimen collected from stress urinary incontinence patients who underwent tension-free vaginal tape. A summary of the sequencing results is shown in Table S1. During quality control with the Seurat R package, the sequencing data of the estimated dead cells and doublet cells were computationally excluded (Table S2), and reduced the batch effect of each sample, the data were integrated with “Harmony”.^10^^,^^11^ The patient demographics, the number of cells analyzed via scRNA-seq, and the pathological information were shown in Figure 1B and Table S3. Unsupervised clustering followed by UMAP revealed 20 distinct clusters (Figure 1C). According to the gene expression of the cell type-specific markers, this cluster analysis broadly segregated the cells into four groups: epithelial cells, immune cells, stromal cells, and endothelial cells (Figure 1D). UMAP plots with cell type-specific markers *(PTPRC* as an immune maker; *KRT18* as an epithelial marker, *COL1A2* as a fibroblast maker, and *CLDN5* as an endothelial marker) showed an obvious segregation of immune, epithelial, stromal, and endothelial lineages (Figure 1D). Among all collected cells, immune cells constituted the largest population, followed by stromal, epithelial, and endothelial compartments (Figure 1E).

### Precise and extensive classification of immune cell lineages in interstitial cystitis

**Figure 2:**
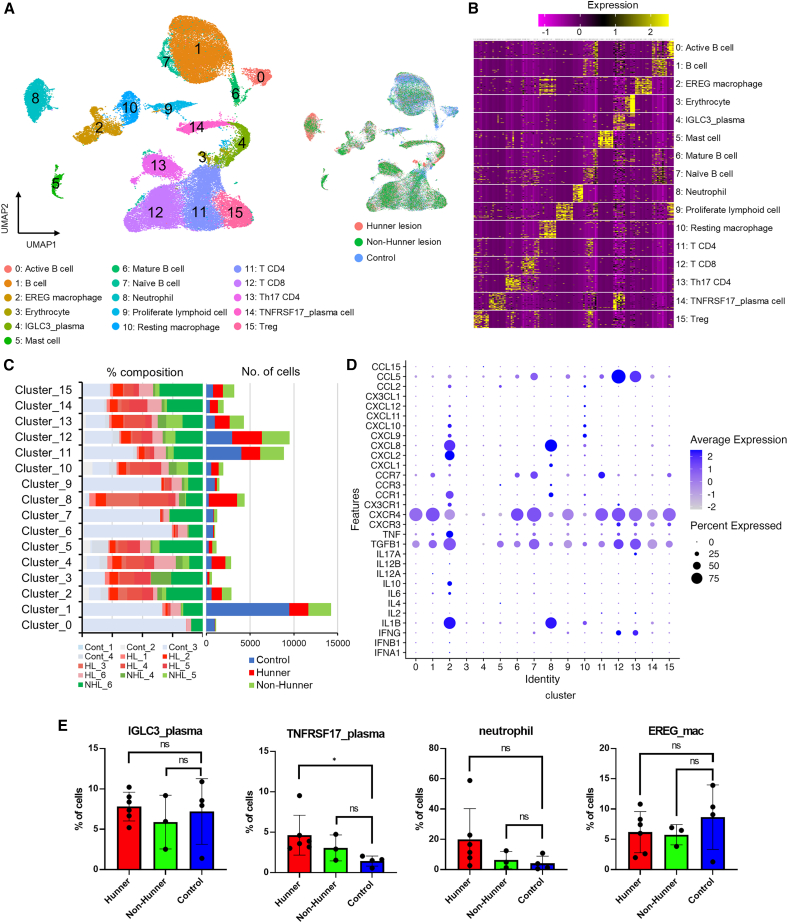
Single-cell transcriptomic analysis of immune components in Hunner lesion, non-Hunner lesions, and non-cystitis controls (A) UMAP plot of immune cells showing clustering results (*k* = 60,729). (B) Heatmap of differentially expressed genes. The average expression level [log_2_(TPM +1)] of selected genes (columns) across each cluster (rows) is displayed. (C) Proportional distribution of immune cells (*x* axis) illustrating the relative abundance of cells from Hunner lesion, non-Hunner lesions, and non-cystitis controls within each cluster (*y* axis). Subsequently, the number of each immune-cell subtype were calculated. Cont indicates controls, HL indicates Hunner lesions, and NHL indicates non-Hunner lesions. (D) Dot plot summarizing the expression profiles of chemokines and their corresponding receptors across immune cell clusters. (E) Comparison of the percentage of plasma cells, neutrophils, and EREG_macrophages among total immune cells across Hunner lesion, non-Hunner lesion, and non-cystitis bladder tissue samples. Data are presented as mean ± SD. Dunnett’s *t* test (∗*p* < 0.05 and ns, no significance).

To investigate immune cell heterogeneity in interstitial cystitis, a total of 60,729 immune cells were reclustered (Figure 2A). The UMAP plot with immune cell lineages revealed 15 clusters. Each cluster of immune cells had distinct differentially expressed genes (Figure 2B and Table S4). Immune cell populations were annotated based on canonical lineage-specific marker genes, including markers for B cells, plasma cells, T cells, myeloid/macrophage populations, neutrophils, mast cells, and proliferating immune cells (Figures S1 and S2). Subclustering analysis further identified distinct B cell, T cell, and myeloid-related subpopulations according to established marker gene expression patterns (Figure S2). The number of cells in each cluster varied among patients; however, in most of clusters, we observed an increase in cell numbers in HIC, both in Hunner lesion and non-Hunner lesion subtypes (Figure 2C). A dot plot was generated to display the clusters with elevated expression of chemokines and their receptors (Figure 2D). While an increase in plasma cells has been previously reported as a pathological feature of HIC,^12^ our scRNA-seq cell annotation identified two distinct plasma cell populations—IGLC3_plasma cells (cluster_4) and TNFRSF11_plasma cells (cluster_14)—that were elevated in the disease group compared to controls (Figure 2C). Notably, the increase of TNFRSF11_plasma was more pronounced in the Hunner lesion subtype (Figure 2E), further validating the robustness of our cell annotation. In our previous studies, we reported an increase in urinary CXCL8 levels in IC patients,^13^ and our current analysis revealed that *CXCL8* expression was elevated in EREG_macrophages and neutrophils (Figure 2D). Specifically, *CXCL8* expression was notably higher in EREG_macrophages within the HIC cohort (Figure S3). Moreover, pathway analysis showed that while there was substantial diversity in immune responses when comparing Hunner lesion to normal tissue, this diversity diminished when comparing Hunner lesions to non-Hunner lesions (Figure S4). Furthermore, both Hunner and non-Hunner lesions demonstrated enrichment of immune- and inflammatory-related signaling pathways, including complement cascade, Fcγ receptor-mediated phagocytosis, and IL-15 signaling pathways, compared with non-cystitis control tissue (Figure S5). These findings suggest that an immune response is occurring more broadly across the bladder in the disease state in not only Hunner lesion but also non-Hunner lesion.

### Precise and extensive classification of fibroblastic lineages in interstitial cystitis

**Figure 3:**
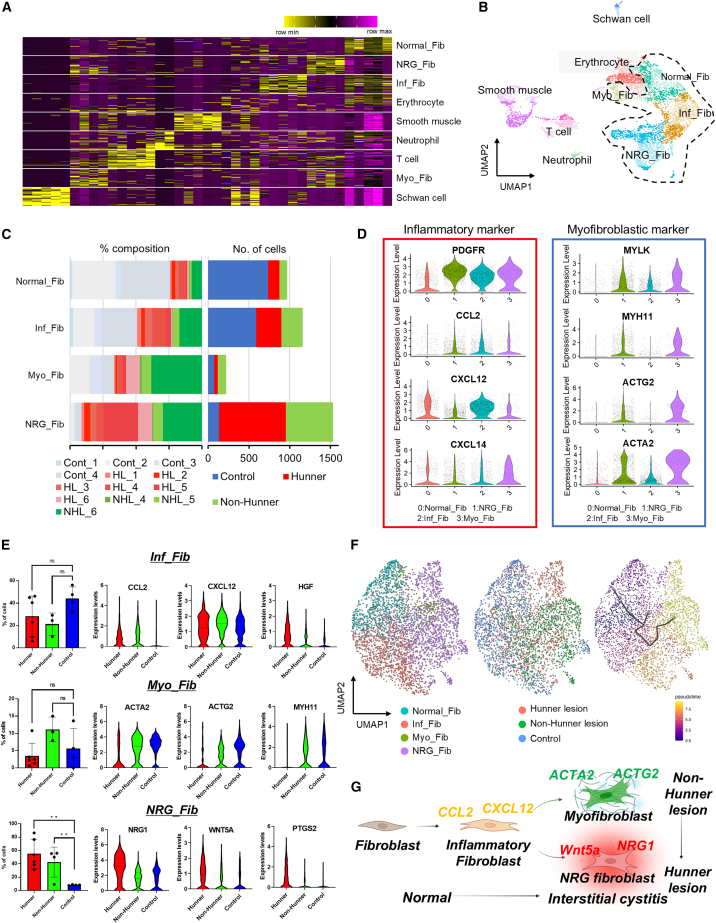
Single-cell transcriptome analysis of the stromal components of Hunner lesion, non-Hunner lesion, and non-cystitis bladder tissue (A) Heatmap of differentially expressed genes. The average expression level [log_2_(TPM +1)] of selected genes (columns) across each cluster (rows) is displayed. (B) UMAP plot of stromal cells showing clustering results (*k* = 6,496). Fibroblast clusters are outlined with dashed lines. (C) Proportional distribution of fibroblasts (*x* axis) illustrating the relative abundance of cells from Hunner lesion, non-Hunner lesions, and non-cystitis controls within each cluster (*y* axis). Subsequently, the number of each stromal cell subtype were calculated. Normal_Fib indicates normal fibroblasts, Inf_Fib indicates inflammatory fibroblasts, Myo_Fib indicates myofibroblasts, and NRG_Fib indicates NRG fibroblasts. Cont indicates controls, HL indicates Hunner lesions, and NHL indicates non-Hunner lesions. (D) Violin plots showing the expression levels of inflammatory and myofibroblastic marker genes across fibroblast clusters. (E) Comparison of the percentages of Inf_Fib, Myo_Fib, and NRG_Fib among fibroblasts across Hunner lesion, non-Hunner lesion, and non-cystitis controls. Violin plots depict the expression differences of representative genes defining each fibroblast subtype. Data are presented as mean ± SD. Dunnett’s *t* test (∗∗*p* < 0.01 and ns, no significance). (F) Pseudotime trajectory analysis illustrating the developmental continuum of fibroblastic lineages. (G) Schematic illustration depicting the proposed model of fibroblast differentiation during disease progression.

To investigate stromal cell heterogeneity in HIC, a total of 6,496 stromal cells were reclustered. Differential gene expression analysis first identified eight transcriptionally distinct stromal cell clusters (Figure 3A and Table S5). These clusters were visualized on a UMAP plot, which demonstrated clear separation of stromal lineages into nine subpopulations (Figure 3B). Fibroblast clusters were annotated based on the expression of canonical marker genes. Normal fibroblasts (Normal_Fib) were annotated using *LUM* and *COL1A1*. Inflammatory fibroblasts (Inf_Fib) were annotated using *CXCL12* and *CXCL14*. Myofibroblasts (Myo_Fib) were annotated using *ACTA2* and *TAGLN* together with fibroblast marker expression. NRG fibroblasts (NRG_fib) were annotated using *NRG1* and *WNT5A*. Smooth muscle cells were identified based on high expression of *ACTA2* and *TAGLN*, together with relatively low expression of fibroblast markers *LUM* and *COL1A1*. Erythrocytes, T cells, neutrophils, and Schwann cells were annotated using *HBB*, *CD3D*, *S100A8*, and *XKR4*, respectively (Figure S6). Within these stromal clusters, we focused in detail on fibroblasts, specifically Normal_Fib, Inf_Fib, Myo_Fib, and NRG_Fib. While Normal_Fib were the most abundant in the control group, the proportions of Inf_Fib, Myo_Fib, and NRG_Fib progressively decreased in control samples but increased within the disease cohort (Figure 3C). Gene ontology (GO) enrichment analysis revealed distinct biological characteristics among fibroblast subtypes. Normal_Fib showed enrichment of translation-related processes, whereas Inf_Fib demonstrated enrichment of cell adhesion-related pathways. Myo_Fib exhibited enrichment of ribosome biogenesis-related pathways, while NRG_Fib showed enrichment of protein catabolic and nucleocytoplasmic transport processes, suggesting that NRG_Fib represents a functionally active fibroblast population rather than a merely structural stromal component (Figure S7). Myofibroblast markers were significantly upregulated in Myo_Fib, while inflammatory markers were elevated in fibroblast subsets other than normal_Fib (Figure 3D). Compared with controls, Myo_Fib tended to increase in non-Hunner and NRG1_Fib was increased in Hunner and non-Hunner lesions (Figure 3E). Genes characteristic of myofibroblasts were highly expressed in non-Hunner lesions, whereas genes specific to NRG_fib were elevated in the disease group, particularly in Hunner lesions (Figure 3E). Notably, *NRG1* and *WNT5A* expression within NRG_Fib was particularly elevated in Hunner lesions, whereas *ACTA2*, a characteristic marker of Myo_Fib, was predominantly enriched in non-Hunner lesions (Figure 3E). Furthermore, trajectory analysis revealed that fibroblasts tended to differentiate toward either Myo_Fib or NRG_fib (Figure 3F). These results suggest that a disease-specific differentiation of fibroblasts occurs in the HIC cohort (Figure 3G).

### Precise and extensive classification of epithelial lineages in interstitial cystitis

**Figure 4:**
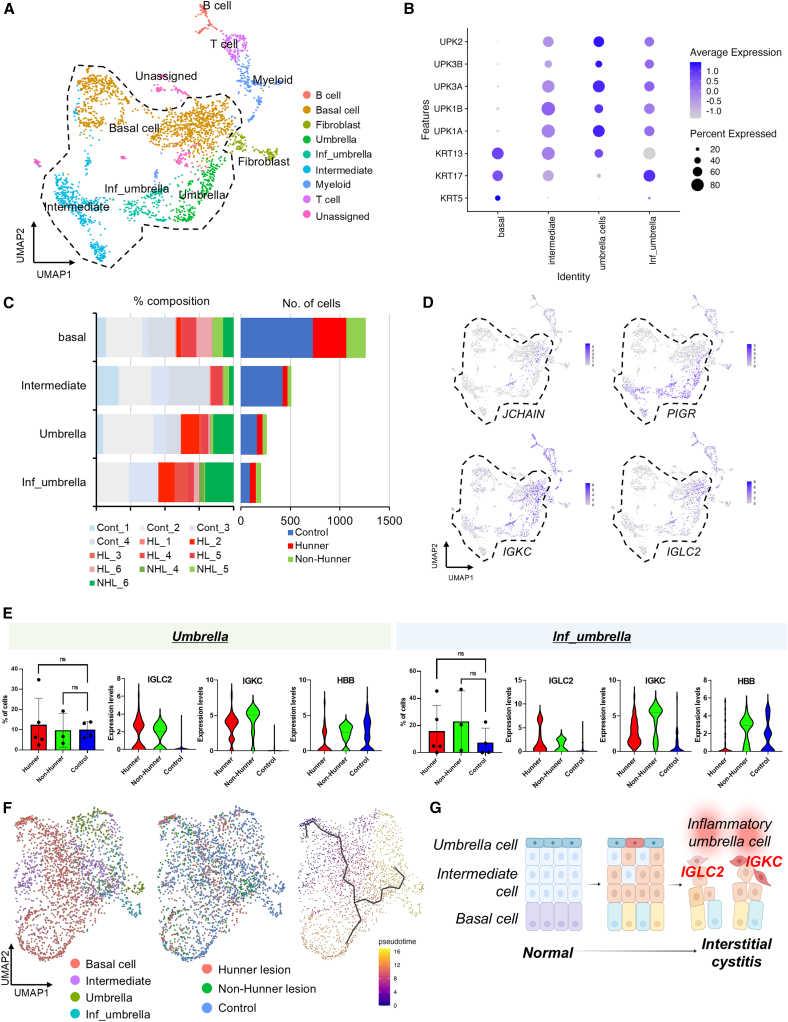
Single-cell transcriptome analysis of the epithelial components of Hunner lesion, non-Hunner lesions, and non-cystitis controls (A) UMAP plot of epithelial cells showing clustering results (*k* = 3,036). Epithelial clusters are outlined with dashed lines. (B) Dot plot of differentially expressed genes. The average expression level [log_2_(TPM +1)] of selected genes (columns) across each cluster (rows) is displayed. (C) Proportional distribution of epithelial cells (*x* axis) showing the relative abundance of cells from Hunner lesion, non-Hunner lesions, and non-cystitis controls within each cluster (*y* axis). Subsequently, the number of each epithelial cell subtype were calculated. Inf_umbrella indicates inflammatory umbrella cells. Cont indicates controls, HL indicates Hunner lesions, and NHL indicates non-Hunner lesions. (D) Representative UMAP plots showing the expression patterns of selected genes. (E) Comparison of the percentages of Umbrella and Inf_umbrella cluster cells among total epithelial cells across Hunner lesion, non-Hunner lesions, and non-cystitis controls. Violin plots depict the expression differences of representative genes defining these two umbrella cell populations. Data are presented as mean ± SD. Dunnett’s *t* test (ns, no significance). (F) Pseudotime trajectory analysis illustrating the developmental progression of epithelial cell lineages. (G) Schematic illustration showing the proposed model of epithelial cell differentiation during disease development.

To investigate epithelial cell heterogeneity in HIC, a total of 3,036 epithelial cells were reclustered (Figure 4A). Differential gene expression analysis first identified eight transcriptionally distinct epithelial cell clusters (Table S6). Feature plots demonstrating representative marker gene expression used for epithelial cell identification and exclusion of non-epithelial populations (Figure S8). The UMAP plot, which included epithelial cell lineages, revealed seven distinct clusters. The characteristic epithelial cell markers for each cluster are shown in the dot plot (Figure 4B). Basal epithelial cells were annotated using canonical basal markers including *KRT5*, *COL17A1*, and *S100A2*. Intermediate epithelial cells were annotated using *ZNF750*, *PDE10A*, and *HS3ST5*. Umbrella cells were annotated using *UPK3A*, *PSCA*, and *SPRR3*. Inflammatory umbrella cells (Inf_umbrella) were annotated using inflammatory epithelial markers including *IDO1*, *LCN2*, *SAA2*, and *MUC1* (Figure 4B and Table S6). A known feature of HIC is the shedding of epithelial cells, which could explain the reduced number of epithelial cells in scRNA-seq analysis, as this method only captures viable cells (Figure 4C). GO analysis of epithelial cell populations revealed that the Inf_Umbrella cluster was enriched for mitochondrial respiration and electron transport chain-related pathways, suggesting metabolic adaptation to inflammatory stress and an activated epithelial state (Figure S9). Previous studies have reported that these cell populations show increased expression in cystitis glandularis characterized by epithelial damage, such as *JCHAIN*, *IGLC2*, *IGKC*, and *PIGR*.^14^ Consistent with these findings, these genes were predominantly expressed within the Inf_umbrella clusters (Figure 4D), and expression of *IGLC2* and *IGKC* was markedly elevated in the HIC cohort compared with controls (Figure 4E). Trajectory analysis further indicated that epithelial cell differentiation ultimately converges on the Inf_umbrella populations (Figure 4F). These results suggest that a disease-specific differentiation of epithelial cell occurs in the HIC cohort (Figure 4G).

### Cellular diversity in the bladder microenvironment

**Figure 5:**
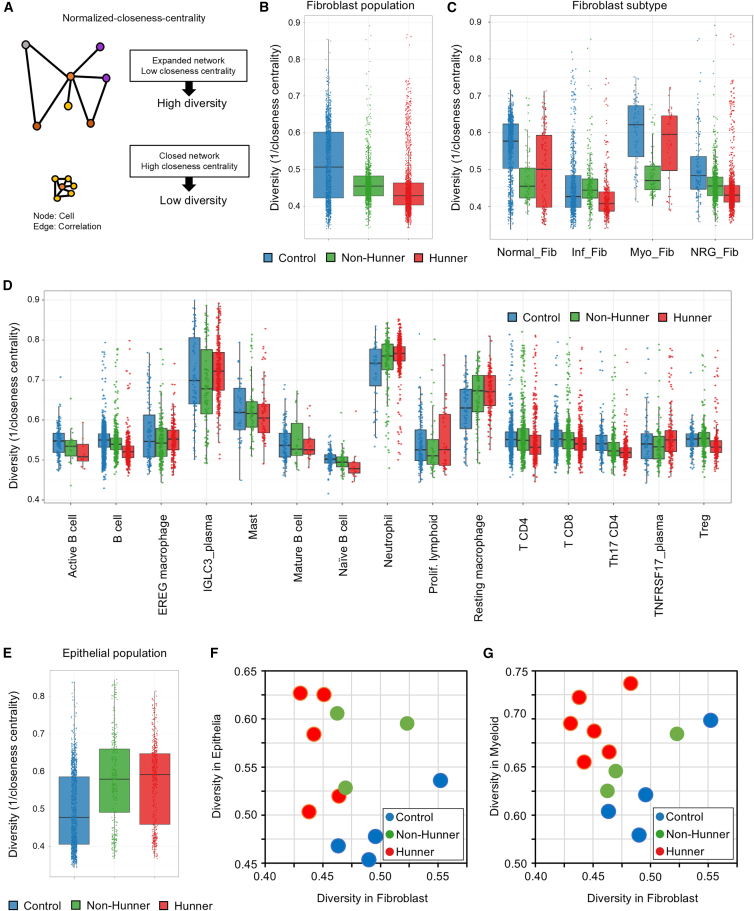
Closeness centrality analysis for elucidating bladder microenvironment heterogeneity (A) Explanation of closeness centrality analysis for quantifying the alterations in gene expression diversity among non-cystitis controls, non-Hunner lesion, and Hunner lesions. With each single cell in the scRNA-seq analysis, the closeness centrality was calculated for all cell types. (B) Boxplots of 1/closeness centrality in total fibroblasts among non-cystitis controls, non-Hunner, and Hunner lesions. Data are presented as median (IQR). (C) Boxplots of 1/closeness centrality in each subtype of fibroblasts among non-cystitis controls, non-Hunner, and Hunner lesions. Data are presented as median (IQR). (D) Boxplots of 1/closeness centrality in each subtype of immune cells among non-cystitis controls, non-Hunner, and Hunner lesions. Data are presented as median (IQR). (E) Boxplots of 1/closeness centrality in total epithelial cells among non-cystitis control, non-Hunner, and Hunner lesions. Data are presented as median (IQR). (F and G) Scatterplots of diversity between two cell types (epithelial cells vs. fibroblasts, F; myeloid cells vs. fibroblasts, G). The Pearson correlation coefficients.

To characterize the breadth of transcriptional variability across cell populations, we applied a previously established diversity-quantification framework based on gene expression topology.^15^ This approach uses closeness centrality as an inverse indicator of heterogeneity: higher centrality reflects more uniform transcriptomic profiles, whereas lower centrality denotes greater diversity within a given population (Figure 5A).

Fibroblast analysis revealed a marked increase in centrality in both Hunner and non-Hunner lesions compared with non-cystitis control tissues, indicating substantially reduced heterogeneity in HIC-associated fibroblasts (Figure 5B). This decrease in diversity was consistently observed across all fibroblast subtypes (Figure 5C). In contrast, immune cell populations—including IGLC3_plasma cells, neutrophils, and resting macrophages—exhibited progressively increased diversity along a gradient from control to non-Hunner and then Hunner lesions (Figure 5D). Epithelial cells showed a similar but more modest trend, with diversity increasing from control bladder tissue to non-Hunner and Hunner lesions (Figure 5E). Within the epithelial compartment, subtype-level analysis demonstrated increased closeness centrality, particularly in Inf_umbrella cells, in HIC samples (Figure S10A).

When visualized in aggregate, these patterns demonstrated that control tissues maintain a heterogeneous fibroblast population alongside relatively homogeneous epithelial states, whereas HIC tissues show diminished fibroblast diversity coupled with pronounced epithelial disorganization (Figure 5F). A comparable shift toward increased heterogeneity was evident when examining myeloid and fibroblast populations together (Figure 5G). Finally, direct comparison of lymphoid and myeloid lineages revealed a clear increase in myeloid cellular diversity in both non-Hunner and Hunner lesions, suggesting progressive immunologic remodeling within the HIC microenvironment (Figure S10B).

### Anomalous cell-to-cell networks in interstitial cystitis

**Figure 6:**
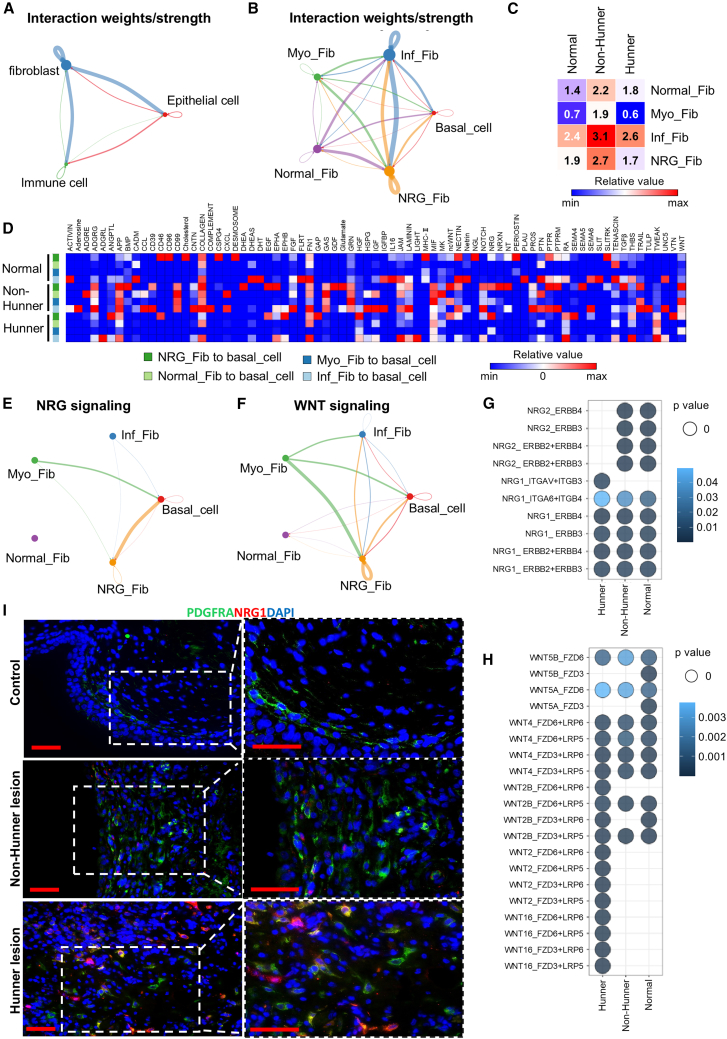
Comparison of interspot interaction signaling among the Hunner lesion, non-Hunner lesions, and non-cystitis controls (A) Interaction strength among fibroblasts, epithelial cells, and immune cells in the overall dataset. (B) Interaction strength among basal cells, Normal_Fib, Myo_Fib, Inf_Fib, and NRG_Fib in the overall dataset. Normal_Fib indicates normal fibroblasts, Myo_Fib indicates myofibroblasts, Inf_Fib indicates inflammatory fibroblasts, and NRG_Fib indicates NRG fibroblasts. (C) Interaction strength from each fibroblast spot to basal spots across Hunner lesion, non-Hunner lesion, and non-cystitis bladder tissue samples. (D) Heatmap of ligand-receptor signaling from each fibroblast subset to basal cell. Columns indicate individual signaling pathways inferred by CellChat, and rows correspond to non-cystitis bladder tissue samples, non-Hunner, and Hunner samples. The color scale represents the relative interaction strength of each pathway. (E and F) Circle plot representing the intercellular communication network within Hunner samples for the NRG signaling pathway (E) and the WNT signaling pathway (F). The thickness of each line represents the interaction strength. (G and H) Dot plot showing the communication probabilities of NRG ligand-receptor signaling (G) and WNT ligand-receptor signaling (H) from NRG_Fib to basal cells in Hunner samples, non-Hunner samples, and non-cystitis bladder tissue samples. Lighter colors indicate higher communication probabilities. (I) Representative immunofluorescence images showing NRG1 and PDGFRA expression in bladder tissue, with co-localization observed in fibroblasts within Hunner and Non-Hunner lesions. Nuclei were counterstained with 4′,6-diamidino-2-phenylindole (DAPI). Scale bars represent 50 μm.

We next examined aberrant intercellular communication among major stromal, epithelial, and immune cell populations. CellChat analysis revealed that, among fibroblast, epithelial, and immune compartments, fibroblasts exhibited the most pronounced outgoing interactions toward epithelial cells (Figure 6A). Further decomposition of fibroblast-derived signaling demonstrated that NRG_Fib and Inf_Fib were the dominant contributors to basal-cell-directed communication (Figure 6B). Notably, these fibroblast-derived cues were markedly amplified in both non-Hunner and Hunner lesions compared with non-cystitis control tissue (Figure 6C). A heatmap summarizing ligand-receptor interactions from each fibroblast subset to basal cells revealed an overall increase in activated signaling pathways in Hunner and non-Hunner lesions (Figure 6D). Among fibroblast subsets, the NRG_Fib population—characterized by elevated *NRG1 and WNT5A* expression and enriched in Hunner lesions—showed particularly enhanced signaling toward basal cells, with prominent activation of NRG and WNT signaling pathways (Figures 6E and 6F). When stromal-epithelial communication was evaluated separately, the NRG1-ITGA6/ITGB4 and WNT5A-FZD6 ligand-receptor axes were identified as selectively activated pathways in both Hunner and non-Hunner lesions (Figures 6G and 6H). Immunohistochemistry confirmed the presence of NRG_Fib, defined by co-expression of NRG1 and PDGFRA specifically within Hunner lesions (Figure 6I). Furthermore, NRG_Fib defined by co-expression of WNT5A and PDGFRA was also specifically detected in Hunner lesions (Figure S11). Together, these findings suggest that NRG_Fib, enriched in Hunner lesions, may contribute to altered stromal-epithelial communication toward basal cells in HIC.

### Validation using public scRNA-seq data

**Figure 7:**
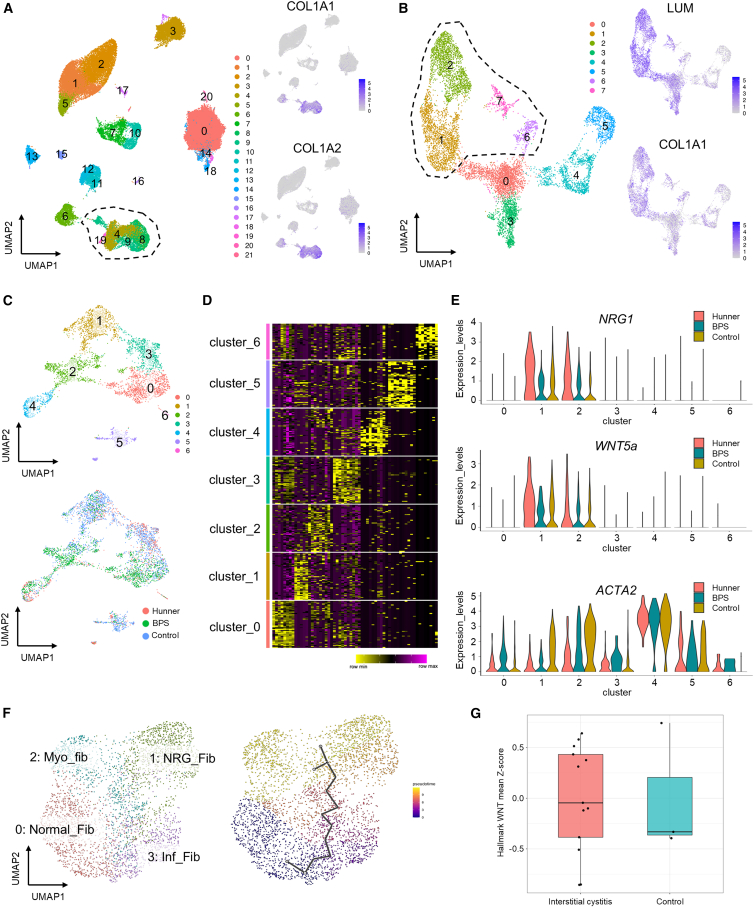
Fibroblast cell dynamics in Hunner lesions revealed by public scRNA-seq data (A) UMAP plot illustrating major cell clusters identified across all bladder samples from the publicly available single-cell RNA-seq dataset GSE175526. Distinct cellular populations were annotated based on canonical marker gene expression. (B) UMAP visualization of cells positive for *LUM* and *COL1A2*, representing the fibroblast-enriched subset extracted for downstream analysis. (C) UMAP plot of fibroblasts showing seven transcriptionally distinct fibroblast clusters, highlighting the heterogeneity of stromal populations within Hunner lesions. (D) Heatmap of cluster-specific differentially expressed genes. The average expression level of representative marker genes is displayed as log_2_(TPM +1) across each fibroblast cluster, illustrating distinct transcriptional programs. (E) Violin plots showing the distribution of expression levels for selected fibroblast-associated genes across the seven clusters, demonstrating cluster-specific enrichment patterns. (F) Pseudotime trajectory analysis depicting the inferred developmental progression of fibroblast subsets. The trajectory highlights potential lineage relationships and dynamic transitions in fibroblast states associated with Hunner lesion pathology. Normal_Fib indicates normal fibroblasts, Myo_Fib indicates myofibroblasts, Inf_Fib indicates inflammatory fibroblasts, and NRG_Fib indicates NRG fibroblasts. (G) Boxplots comparing WNT signaling score between interstitial cystitis (red) and non-cystitis controls (blue) from the publicly available bulk RNA-seq dataset GSE57560. Median, IQR, and individual data points are shown.

To independently validate these findings, we analyzed a publicly available bladder single-cell RNA-seq dataset (GSE175526) and reconstructed fibroblast subsets using the same analytical framework. All bladder cells positive for *COL1A1* and *COL1A2* were extracted, and a *de novo* UMAP embedding was generated (Figure 7A). Fibroblasts were further refined based on the expression of canonical markers (*COL1A1* for fibroblasts, *CD3D* for lymphocytes, *LYZ* for myeloid cells, *CLDN5* for endothelial cells, *DES* for smooth muscle, *SLC6A1* for neural cells, and *CCL21* for lymphatic endothelial cells), yielding a transcriptionally coherent stromal population (Figures 7B and S12). Unsupervised clustering identified seven molecularly distinct fibroblast subsets, and cells originating from Hunner lesions were distributed among these populations (Figure 7C). A heatmap highlighted key signature genes defining each subset (Figure 7D and Table S7). Based on the expression of lineage-specific markers, cluster 4 was considered to represent epithelial cell contamination, characterized by expression of *S100A2*, *KRT8*, and *KRT19*; cluster 5 represented T cell contamination, marked by *CD2*, *CD3D*, and *CD3E* expression; and cluster 6 was interpreted as Schwann cell contamination based on *NGFR* and *CDH19* expression. In addition, based on the expression of inflammatory fibroblast-associated genes including *TNFAIP6*, *LIF*, and *CXCL2*, cluster 3 was annotated as Inf_Fib (Table S7). *NRG1* and *WNT5A* expression was highly enriched in cluster 1—particularly within fibroblasts derived from Hunner lesions—while *ACTA2* was predominantly expressed in cluster 2 (Figure 7E). Accordingly, clusters 1, 2, and 0 were classified as NRG_Fib, Myo_Fib, and Normal_Fib, respectively. Trajectory inference using clusters 0–3 demonstrated a bifurcating lineage pattern, with fibroblasts progressing toward either an NRG-enriched (cluster 1) or myofibroblastic (cluster 2) state (Figure 7F). Consistent with these single-cell findings, analysis of a bulk RNA-seq dataset demonstrated elevated WNT signaling activity in tissues from Hunner lesions, further supporting activation of this pathway in the disease microenvironment (Figure 7G). Importantly, this NRG_Fib population was consistently detected in Hunner lesion-derived cells in the re-analysis of the public dataset, supporting its reproducible involvement in disease-associated stromal remodeling.

## Discussion

Despite the substantial impact of HIC on patients’ quality of life, therapeutic options remain limited because its underlying pathophysiology remains poorly defined. Using scRNA-seq, we delineated the cellular landscape of HIC by analyzing samples from Hunner lesions, non-Hunner lesions, and non-cystitis controls. This multi-regional sampling enabled the identification of disease-wide alterations in both immune and stromal compartments. Focusing on fibroblasts—an understudied cell type in HIC—we identified a subset of fibroblasts, termed NRG_Fib. Heterogeneity analysis further revealed distinct lineage-specific patterns: fibroblast heterogeneity decreased with disease progression, whereas epithelial heterogeneity increased, reflecting the accumulation of tissue injury. In the immune compartment, heterogeneity increased in selected populations, particularly IGLC3_plasma cells, indicating an expanded and diversified humoral response in HIC.

Previous scRNA-seq studies on interstitial cystitis have predominantly focused on immune cell subsets. Su et al. reported an enrichment of M2/M2-like macrophages as a hallmark of HIC, suggesting an anti-inflammatory yet tissue-remodeling immune milieu.^9^ Similarly, Peng et al. combined scRNA-seq with spatial transcriptomics to demonstrate that immune subpopulations in HIC bladders are localized near fibroblasts and the urothelial region, in contrast to their distribution around smooth muscle cells in controls.^8^ Our findings extend these observations by demonstrating that both Hunner and non-Hunner regions exhibit pronounced immune activation and complex intercellular signaling, confirming that HIC represents a pan-bladder inflammatory disorder rather than a lesion-confined pathology. Although previous studies highlighted immune activation, they did not systematically evaluate epithelial or stromal compartments.^8^^,^^9^

A distinctive feature of our study is the in-depth analysis of interstitial cell populations, particularly fibroblasts, which have recently emerged as key regulators of chronic inflammation and tissue remodeling.^16^ Through trajectory and clustering analyses, we identified two fibroblast subsets—NRG_Fib and Myo_Fib—that expand preferentially in HIC. NRG_Fib were enriched within Hunner lesions, while Myo_Fib predominated in non-Hunner regions, suggesting spatially distinct fibroblast activation states. Cell-cell interaction analysis further revealed that NRG_Fib strongly signal to basal epithelial cells, implicating fibroblast-driven epithelial modulation as a central mechanism in Hunner lesion formation. In addition to stromal remodeling, we characterized the differentiation trajectory of urothelial cells, revealing that basal cells give rise to Inf_umbrella cells. The upregulation of *IGLC2* and *IGKC* in both Hunner and non-Hunner lesions suggests an inflammatory response-driven epithelial remodeling process. Similar epithelial phenotypes have been described in cystitis glandularis, a chronic hyperplastic bladder condition,^14^ further supporting a shared inflammatory epithelial response program.

Heterogeneity analysis revealed lineage-specific patterns across fibroblast, epithelial, and immune compartments in Hunner and non-Hunner lesions. Fibroblasts showed overall convergence of heterogeneity, yet diverged phenotypically, with NRG_Fib enriched in Hunner lesions and Myo_Fib predominant in non-Hunner lesions, indicating region-specific differentiation within the same disease entity. By contrast, epithelial cells exhibited increased heterogeneity in both lesion types, consistent with progressive epithelial injury. Notably, IGLC3_plasma cells demonstrated increased heterogeneity across both lesions, suggesting expansion of diverse antigen-driven humoral responses. This is in line with histopathological evidence of robust B cell activation characteristic of Hunner-type disease,^17^ λ light chain-restricted B cell expansion,^6^ and dense plasma cell infiltration.^18^ Given that *IGLC3* encodes a mucosa-associated λ constant region, the increased heterogeneity of IGLC3_plasma cells in our dataset likely reflects this heightened immunological activity.

Our ligand-receptor interaction analysis using CellChat identified fibroblast-to-basal epithelial cell signaling as one of the most dominant intercellular communication axes. Further stratification of fibroblasts revealed that, compared with controls, multiple fibroblast subtypes in both Hunner and non-Hunner lesions exhibited increased signaling toward basal epithelial cells. Focusing on NRG_Fib—markedly enriched in Hunner lesions—we found that pathways involving NRG1 and WNT5A as fibroblast-derived ligands were selectively upregulated when basal epithelial cells were designated as receptor populations. Moreover, NRG_Fib showed particularly high expression of NRG1 and WNT5A within Hunner lesions. Notably, WNT5A has been reported to enhance IFN-γ-driven IL-12 production in dendritic cells, promoting Th1 differentiation in colitis.^19^ Together with previous reports describing EBV-associated inflammatory signaling and epithelial immune interactions in HIC,^6^^,^^20^ our findings further support a potential role for fibroblast-epithelial crosstalk in orchestrating chronic inflammation in HIC. Although further investigation is warranted, these findings suggest that fibroblast-centered signaling pathways, particularly NRG1- and WNT5A-mediated interactions, may play a pivotal role in epithelial remodeling and immune activation within the HIC microenvironment.

In summary, our single-cell transcriptomic analysis delineates the cellular architecture and intercellular signaling networks underlying HIC. We demonstrate that HIC is characterized by global immune activation across the bladder and by the emergence of disease-associated fibroblast subsets, particularly NRG_Fib. Evaluation of cellular heterogeneity further revealed lineage-specific patterns: fibroblast heterogeneity decreased with disease progression, whereas epithelial heterogeneity increased, consistent with cumulative tissue injury. Within the immune compartment, the heterogeneity of IGLC3_plasma cells increased in both non-Hunner and Hunner lesions, reflecting heightened and diversified immunological activity. Together, these findings provide an integrated view of immune and non-immune cellular dynamics in HIC and reveal disease-associated remodeling of stromal and epithelial populations.

### Limitations of the study

This study has several limitations that warrant acknowledgment. First, as HIC is a rare disease, our cohort size was limited. Second, control tissues were obtained from patients with stress urinary incontinence undergoing tension-free vaginal tape surgery and may not fully represent healthy bladder mucosa. Nevertheless, this approach enabled ethical and practical tissue collection while providing meaningful disease-control comparisons. Third, ambient RNA contamination is an inherent limitation of droplet-based single-cell RNA sequencing analyses. Although computational correction methods such as CellBender were not applied, immunohistochemical validation supported the stromal localization of NRG1/WNT5A-positive fibroblasts, reducing the likelihood that these findings were solely attributable to ambient RNA contamination. Finally, although we identified NRG_Fib enrichment in Hunner lesions and associated stromal-epithelial signaling pathways, the biological functions and mechanistic contributions of these fibroblast populations to HIC pathogenesis remain to be determined.

## Resource availability

### Lead contact

Further information and requests for resources should be directed to and will be fulfilled by the lead contact, Fumihiko Urabe (furabe0809@gmail.com).

### Materials availability

This study did not generate new unique reagents.

### Data and code availability

• The raw sequencing data have been deposited in the DDBJ Sequence Read Archive (DRA) under accession numbers DRR895704–DRR895726, and processed count matrices have been deposited in the Genomic Expression Archive (GEA) under accession number E-GEAD-1192.
• This study did not generate custom code. Analyses were performed using publicly available software packages, including Seurat (v4.3.3), Harmony, DoubletFinder, CellChat, ClusterProfiler, and GraphPad Prism. Additional information regarding software and analytical parameters is provided in the STAR Methods section.
• Any additional information required to reanalyze the data reported in this paper is available from the lead contact upon request.

## Acknowledgments

The authors thank Textcheck Inc. for providing English language editing services for this manuscript. This study was funded by a Grant-in-Aid for Scientific Research, the 10.13039/501100001691Japan Society for the Promotion of Science (JSPS KAKENHI grant no 24K12448), 10.13039/100007449Takeda Science Foundation, 10.13039/100015542GSK Japan, the 10.13039/501100008880Kanae Foundation for the Promotion of Medical Science, 10.13039/100018036Nippon Shinyaku Co., Ltd., and 10.13039/100008732The Uehara Memorial Foundation. The funders played no role in study design, data collection, analysis and interpretation of data, or the writing of this manuscript. We also appreciate the artwork provided by MEDICAL FIG., a service of Medical Education, Inc.

## Author contributions

Conceptualization, F.U. and Y.Y.; methodology, F.U. and J.N.; investigation, F.U., K.Y., Y.H., N.W., and H.S.; formal analysis, F.U. and K.Y.; data curation, F.U. and K.Y.; writing – original draft, F.U.; writing – review and editing, all authors; supervision, T.K. and Y.Y.; funding acquisition, F.U. and Y.Y.

## Declaration of interests

The authors declare no competing interests.

## STAR★Methods

### Key resources table

**Table:** 

REAGENT or RESOURCE	SOURCE	IDENTIFIER
**Antibodies**		

Rabbit anti-PDGF Receptor α (D1E1E) XP	Cell Signaling Technology	Cat#3174
Mouse anti-Neuregulin-1 (E-12)	Santa Cruz Biotechnology	Cat#sc-393006
Rat anti-WNT5A	R&D Systems	Cat#AF645

**Biological samples**		

Human Hunner lesion bladder biopsy specimens	This study	IRB #32-405(10494)
Human non-Hunner lesion bladder biopsy specimens	This study	IRB #32-405(10494)
Human non-cystitis control bladder biopsy specimens	This study	IRB #32-405(10494)

**Critical commercial assays**		

Chromium Next GEM Single Cell 3′ GEM Library & Gel Bead Kit v3.1	10x Genomics	PN-1000121
Chromium Next GEM Chip G Single Cell Kit	10x Genomics	PN-1000120
Multi Tissue Dissociation Kit 1	1Miltenyi Biotec	Cat#130-110-201

**Deposited data**		

Raw scRNA-seq data	DDBJ Sequence Read Archive (DRA)	DRR895704–DRR895726
Processed count matrices	Genomic Expression Archive (GEA)	E-GEAD-1192
Public scRNA-seq dataset	Gene Expression Omnibus (GEO)	GSE175526
Public bulk RNA-seq dataset	Gene Expression Omnibus (GEO)	GSE57560

**Software and algorithms**		

Cell Ranger v6.1.2	10x Genomics	https://www.10xgenomics.com
R	https://www.r-project.org/	https://www.r-project.org/
Seurat v4.3.3	Satija Lab	https://satijalab.org/seurat
Harmony	Korsunsky et al.^10^	https://github.com/immunogenomics/harmony
DoubletFinder	McGinnis et al.^21^	https://github.com/chris-mcginnis-ucsf/DoubletFinder
CellChat	Jin et al.^22^	https://github.com/sqjin/CellChat
ClusterProfiler	Bioconductor	https://bioconductor.org
Ingenuity Pathway Analysis (IPA)	QIAGEN	https://digitalinsights.qiagen.com
GraphPad Prism v8	GraphPad Software	https://www.graphpad.com
Morpheus	Broad Institute	https://software.broadinstitute.org/morpheus/

### Experimental model and study participant details

#### Patients and disease specimens

We collected six samples from Hunner lesions in six patients with HIC (five females and one male). Additionally, samples were collected from non-Hunner lesions in three of these patients, including one male and two female patients. This sex distribution reflects the known female predominance of HIC. Representative imaging findings are shown in Figure S13. For comparison, four control bladder tissue samples were obtained from female non-cystitis control individuals undergoing tension-free vaginal tape surgery for stress urinary incontinence; control tissues were primarily collected from the posterior bladder wall. The diagnosis of HIC was established using the East Asian guidelines criteria.^1^ Informed consent was obtained from all biopsy participants as part of an approved ongoing research protocol by the ethical committee of The Jikei University School of Medicine (#32-405(10494)). Written informed consent was received from the patients. Bladder biopsy was performed using the cold cup biopsy technique. Isolated bladder tissue biopsy samples were mechanistically minced and dissociated with enzymes according to a Multi Tissue Dissociation Kit 1 protocol (Catalog no. 130-110-201, Miltenyi Biotec). After the dissociation, viability of single cells was confirmed >90% by trypan blue staining. Cells were then washed in PBS supplemented with 0.1% BSA (Sigma-Aldrich), passed through 70-μM and 40-μM filters, centrifugated at 300 x g for 10 minutes, and resuspended in PBS supplemented with 1% BSA.

### Method details

#### Chromium 10x Genomic library preparation and sequencing

Cells were counted and resuspended in PBS supplemented with 0.04% BSA for loading for single-cell library construction on the 10x Genomic platform (10x Genomics, CA, USA). All experimental samples were analyzed with a Chromium Next GEM single Cell 3′ GEM, Library & Gel Beads Kit v.3.1 and Chromium Next GEM Chip G single Cell Kit according to the manufacturer’s instructions in the Chromium Next GEM Single 3′ Reagent Kit V.3.1 User Guide. Briefly, approximately 5,000 cells were loaded into each channel and were then partitioned into gel beads in emulsion in a Chromium Controller for cell lysis and barcoded reverse transcription of RNA, followed by amplification, fragmentation, and 5′ adaptor and sample indexing. Sequencing was performed on the HiSeq platform (Illumina, CA, USA) with an average sequencing read of 140,486 reads/cell.

#### Single-cell RNA sequencing data alignment

RAW sequencing data were processed with the Cell Ranger pipeline (version 6.1.2; 10 X Genomics) and mapped to the human genome (GRCh38-2020-A) to generate matrices of gene counts by cell barcodes.

#### Data quality control and normalization

The scRNA-seq analyses, including normalization, scaling, clustering of the cells, and identifying cluster marker genes, were performed using the R software package Seurat version 4.3.3. We extracted single cells with nFeature_RNA >500, nFeature_RNA <8,000, and percent.mt < 20 to exclude low-quality or dying cells. No additional UMI/count-based filtering threshold was applied. Doublet detection was performed on each sample independently using DoubletFinder.^21^ The optimal pK value for each sample was determined by BCmetric optimization, and the expected doublet rate was set according to the number of cells in each sample. After doublet removal, only singlet cells were integrated into a combined Seurat object. Subsequently, log normalization was performed using the function “NormalizeData” in Seurat. Samples were log-normalized and scaled for the number of genes and percentage of mitochondrial reads.

#### Data clustering and dimensionality reduction

We performed principal component analysis for dimensionality reduction in R with Seurat. To minimize the difference in batch effects, scRNA-seq data were integrated with Harmony.^10^^,^^11^ Clustering of a single cell was performed by the functions “FindNeighbors” and “FindClusters” from Seurat. The dimensionality-reduced cell clustering is shown as a uniform manifold approximation and projection (UMAP) plot by the function ‘run UMAP’. Clusters were grouped based on expression of tissue compartment markers (for example, *KRT18* (epithelial intermediate filament protein) for epithelial cells, *COL1A2* (collagen type 1 alpha 2 chain) for stromal cells, *PTPRC* (protein tyrosine phosphatase receptor type C) for immune cells, *CLDN5* (claudin5) for endothelial cells). After the initial clustering, major cellular compartments, including immune, stromal, and epithelial cells, were subsetted and reanalyzed independently to achieve higher-resolution clustering and finer cell-type annotation within each lineage. Cell-type annotation was performed using canonical marker genes with reference to previously published single-cell RNA sequencing studies and atlases of immune and stromal cell populations, including inflammatory fibroblasts, intratumoral B cells, and T-cell states.^23^^,^^24^^,^^25^^,^^26^^,^^27^ Heatmaps were generated by Morpheus software from the Broad Institute (https://software.broadinstitute.org/morpheus/). IPA (Ingenuity Pathway Analysis; https://ww.digital-biology.co.jp/allianced/products/ipa/) was used to analyze transcriptomic data for predicting enriched canonical pathways and upstream regulators.

#### Pathway enrichment analysis

We performed enrichment analysis with the signature gene list from each epithelial, immune, and stromal cell cluster using the ‘ClusterProfiler’ packages in R.^28^ For enrichment analysis, gene symbols were converted to ENTREZ IDs using the ‘org.Hs.eg.db’ package Carison M, R package version 3.10.0., 2019). Gene Ontology (GO) enrichment analysis using the ‘enrichGO’ function was performed by the BH method.

#### CellChat analysis

We extracted normalized expression data from a Seurat object and created a CellChat object using the “createCellChat” function.^22^ For ligand–receptor interaction data, we utilized the CellChatDB and designated “Secreted Signaling” as the analysis category using the “subsetDB” function. Next, we performed data preprocessing with the “identifyOverExpressedGenes” and “identifyOverExpressedInteractions” functions. Communication probabilities between annotated cell populations were estimated using the “computeCommunProb” function. In this analysis, we applied type = “truncatedMean” and trim = 0.1. To ensure the reliability of the results, we used the “filterCommunication” function and set min.cells = 10. Following these steps, pathway-specific communication probabilities were calculated using the “computeCommunProbPathway” function, and the overall intercellular communication network was aggregated using the “aggregateNet” function.

#### Heterogeneity score calculation

We calculated correlation coefficients to evaluate closeness centrality in gene expression within each cell population, following previously described procedures.^15^^,^^29^ The heterogeneity score was defined as the inverse of this centrality.

#### Public single cell RNA-seq and bulk RNA-seq data analysis

Publicly available single-cell RNA-seq data were retrieved from the GEO dataset GSE175526, which includes samples from patients with HIC (n= 2), BPS (n= 3), and non-cancerous bladder tissues from individuals with bladder cancer (n= 2), the latter serving as normal controls. Quality control was performed by calculating mitochondrial gene percentages using the “PercentageFeatureSet” function. We extracted single cells with nFeature_RNA >500, nFeature_RNA <8,000, and prercent.mt < 20 to exclude low-quality cells. Doublet detection was performed on each sample independently using DoubletFinder. After doublet removal, each sample was merged into a Seurat object and normalized using “NormalizeData”. Highly variable genes were identified with “FindVariableFeatures”, followed by data scaling and PCA. Batch effects were corrected using Harmony integration, and UMAP dimensionality reduction was applied for visualization. Cell type annotation was performed based on the expression patterns of the fibroblast marker genes *COL1A1* and *COL1A2*. This dataset was used to validate fibroblast populations that were identified as characteristic of Hunner lesions in our analysis. This dataset was used to validate fibroblast populations that were identified as characteristic of Hunner lesions in our analysis. For external validation of pathway activity at the bulk-transcriptome level, we additionally analyzed a publicly available bulk RNA-seq cohort (GSE57560), comprising 16 samples (interstitial cystitis, n = 13; normal controls, n = 3). The Hallmark_WNT_BETA_CATENIN_SIGNALING gene set was obtained from the Molecular Signatures Database (MSigDB) using the msigdbr R package (version 25.1.0). For each sample, we calculated a WNT signaling score by computing the mean Z-score across the 42 genes included in the Hallmark signature. Differences in WNT signaling activity between interstitial cystitis and control tissues were assessed using the Wilcoxon rank-sum test, and results were visualized with the ggplot2 package (version 3.5.2).

#### Immunofluorescence staining

The tissue samples were fixed in formalin and embedded in paraffin. Following dewaxing and rehydration, heat-induced epitope retrieval was performed by boiling the specimens in 1/200 diluted ImmunoSaver (Wako, 097-06192) at 98°C for 45 min. The specimens were treated with 0.1% Triton X-100 for tissue permeabilization. After treatment with a Protein Block Serum-Free blocking reagent (DAKO, Code X0909) at room temperature for 30 min, the specimens were incubated with primary antibodies at 4°C overnight. They were then incubated with Alexa Fluor fluorescent secondary antibodies for 1 hour at room temperature. Images were captured using a BZ-X700 microscope (Keyence Corporation). Antibodies used were rabbit anti-PDGF Receptor α (D1E1E) XP(R) (Cell Signaling Technology, 3174), mouse anti-Neuregulin-1 (Santa Cruz Biotechnology, E-12), and rat anti-WNT5A (R&D Systems, AF645).

### Quantification and statistical analysis

Statistical analyses were performed using GraphPad Prism version 8 (GraphPad Software, San Diego, CA, USA) unless otherwise specified. Statistical details for each experiment, including the statistical tests used, exact sample sizes (n), definitions of center and dispersion, and significance levels, are provided in the corresponding figure legends, figures, results section, or supplementary materials.

For comparisons among three groups, one-way analysis of variance (ANOVA) followed by Dunnett’s multiple comparisons test was used. For analysis of the public bulk RNA-seq dataset (GSE57560), differences between groups were assessed using the Wilcoxon rank-sum test. Correlations between diversity scores were evaluated using Pearson correlation analysis.

For scRNA-seq analyses, n represents the number of biological samples unless otherwise indicated. Specifically, six Hunner lesion samples, three non-Hunner lesion samples, and four non-cystitis control samples were analyzed. Cell numbers included in each analysis are reported in the corresponding figures, figure legends, and Supplementary Tables. Data are presented as mean ± SD for group comparisons and as median with IQR for closeness centrality analyses, as indicated in the corresponding figure legends. Statistical significance was defined as a *P* value <0.05.

### Additional resources

This study did not involve a clinical trial.

## Supplemental information

Document S1. Figures S1–S13

Table S1. scRNA-seq information of each sample

Table S2. Number of cells excluded from downstream analysis in each sample

Table S3. Summary of patient information

Table S4. Upregulated 10 genes of all clusters for immune cells

Table S5. Upregulated 5 genes of all clusters for stromal cells

Table S6. Upregulated 5 genes of all clusters for epithelial cells

Table S7. Upregulated 10 genes of all clusters for fibroblast in public dataset
