# Supplementary material for: Single-cell transcriptomics reveals bladder microenvironment dynamics in Hunner type interstitial cystitis
Source: iScience. 2026 Jul 9;29(8):116725. doi: 10.1016/j.isci.2026.116725 (PMC13380760; doi:10.1016/j.isci.2026.116725)
Supplement: Document S1. Figures S1–S13 [file mmc1.pdf]

iScience, Volume *29*

Supplemental information

Single-cell transcriptomics reveals bladder microenvironment dynamics in Hunner type interstitial cystitis

Fumihiko Urabe, Kentaro Yoshihara, Jun Nakayama, Yuta Hirano, Naoaki Watanabe, Hironori Suzuki, Miyaka Umemori, Kagenori Ito, Taro Igarashi, Shun Sato, Takahiro Kimura, Akira Furuta, and Yusuke Yamamoto

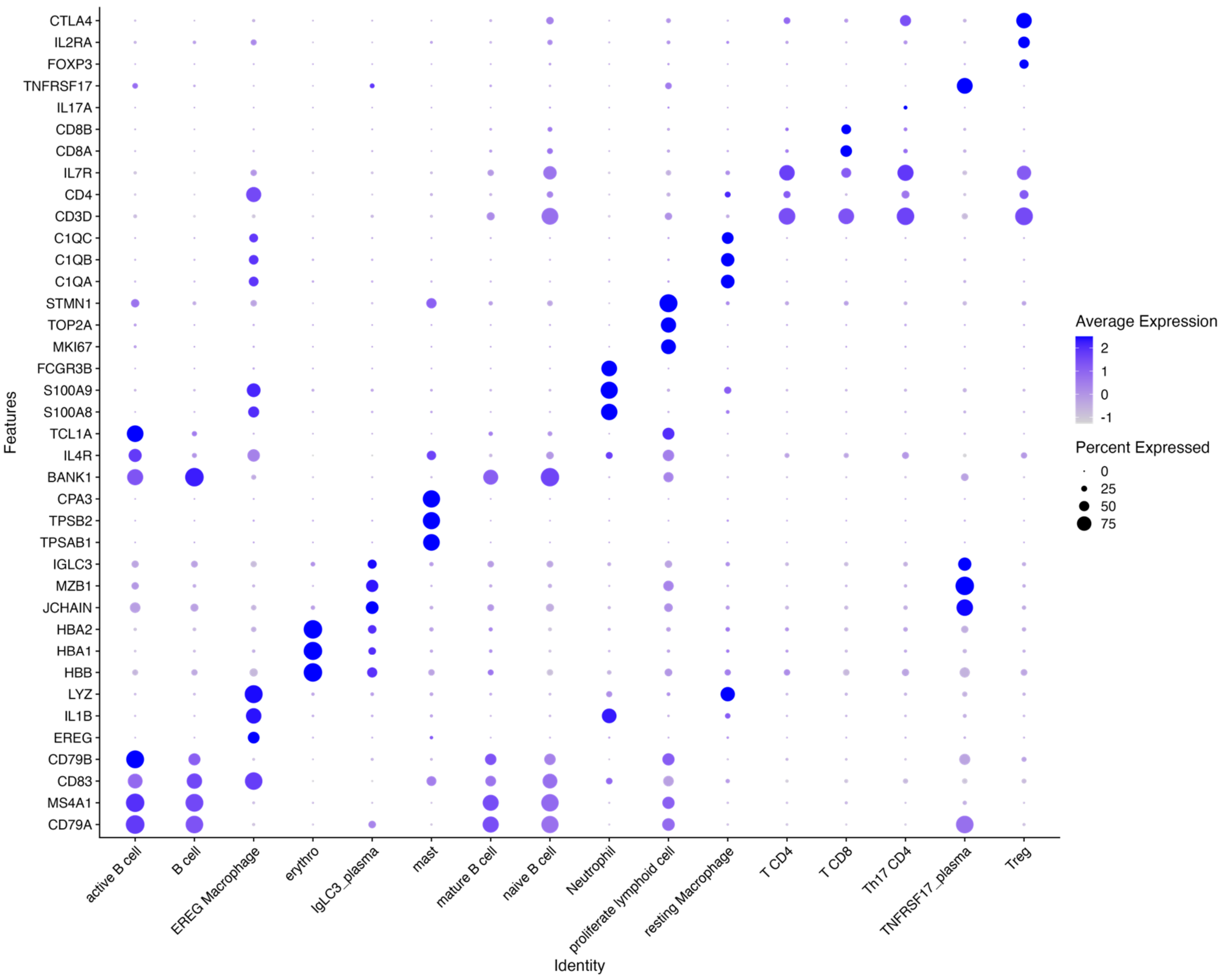

Figure S1. Dot plot of canonical marker gene expression across annotated immune cell clusters. Dot plot showing representative marker gene expression used for immune cell cluster annotation.
The size of each dot represents the proportion of cells expressing the indicated gene, whereas the color intensity represents the scaled average expression level.
Immune cell clusters were annotated based on canonical lineage-specific markers, including *CD79A*, *CD79B*, *MS4A1*, *BANK1*, *IL4R*, and *TCL1A* for B cells; *JCHAIN*, *MZB1*, *IGLC3*, and *TNFRSF17* for plasma cells; *LYZ*, *EREG*, *IL1B*, *C1QA*, *C1QB*, and *C1QC* for myeloid cells/macrophages; *S100A8*, *S100A9*, and *FCGR3B* for neutrophils; *TPSAB1*, *TPSB2*, and *CPA3* for mast cells; *MKI67*, *TOP2A*, and *STMN1* for proliferating immune cells; *CD3D*, *CD4*, *IL7R*, *CD8A*, and *CD8B* for T cells; *IL17A* for Th17 cells; and *FOXP3*, *IL2RA*, and *CTLA4* for regulatory T cells.

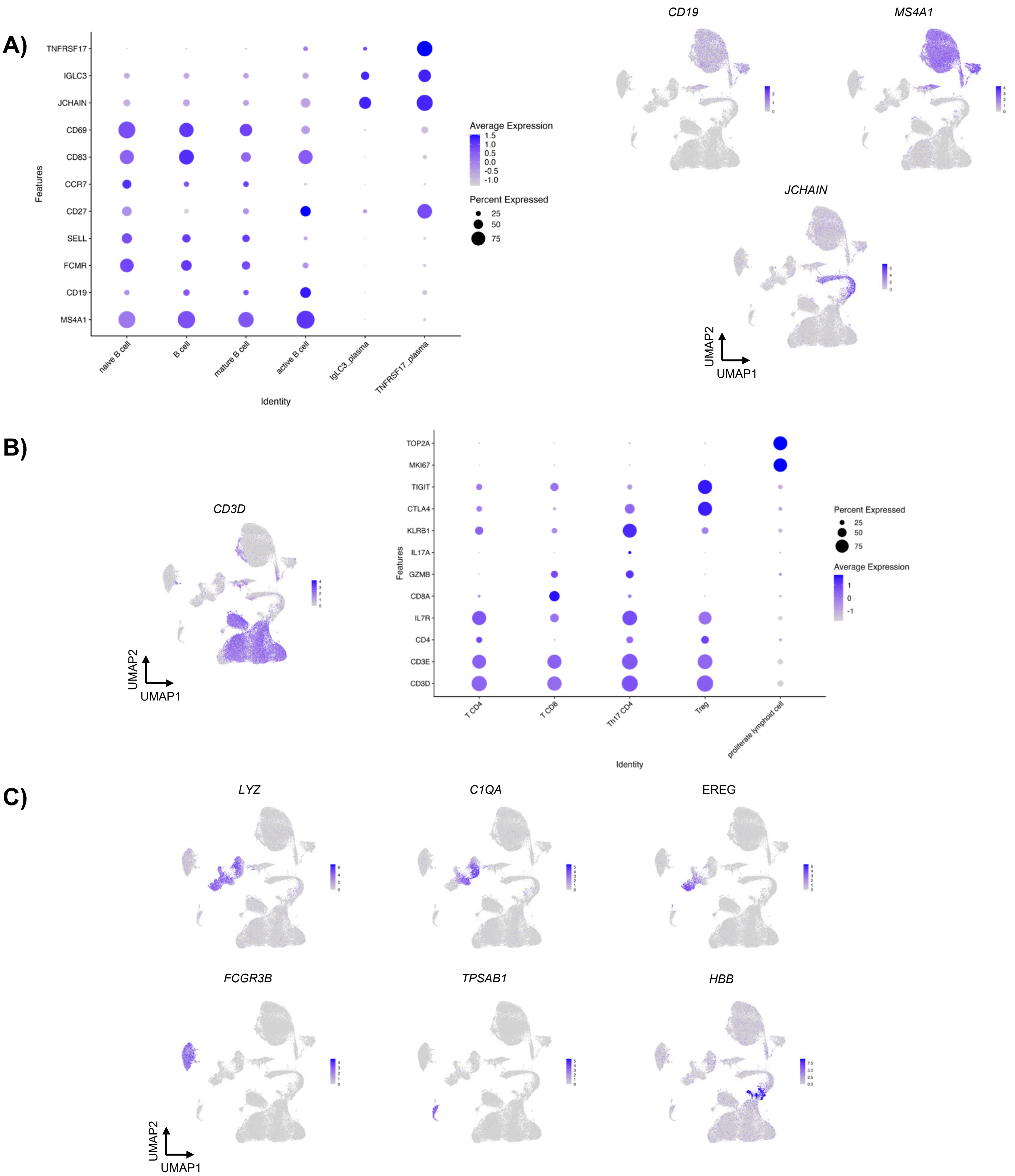

Figure S2. Immune cell subclassification by canonical marker genes.

A) B-cell and plasma-cell subclusters. Dot plot showing representative marker gene expression across B-cell clusters. Dot size indicates the proportion of expressing cells, and color intensity indicates scaled average expression. UMAP feature plots show *CD19*, *MS4A1*, and *JCHAIN* expression. Naive B cells were identified by *FCMR*, *SELL*, and *CCR7*; mature B cells by *CD27* and *BANK1*; active B cells by *CD83* and *CD69*; and plasma cells by *JCHAIN*, *IGLC3*, and *TNFRSF17*.

B) T-cell subclusters. Dot plot showing representative marker gene expression across T-cell clusters. UMAP feature plots show *CD3D* expression. CD4 T cells were identified by *CD4* and *IL7R*, CD8 T cells by *CD8A* and *GZMB*, Th17 CD4 cells by *IL17A* and *KLRB1*, regulatory T cells by *CTLA4* and *TIGIT*, and proliferating lymphoid cells by *MKI67* and *TOP2A*.

C) Myeloid and other immune cell populations. UMAP feature plots showing representative marker gene expression, including *LYZ*, *C1QA*, *EREG*, *FCGR3B*, *TPSAB1*, and *HBB*, for identification of myeloid/macrophage, neutrophil, mast cell, and erythroid-related populations.

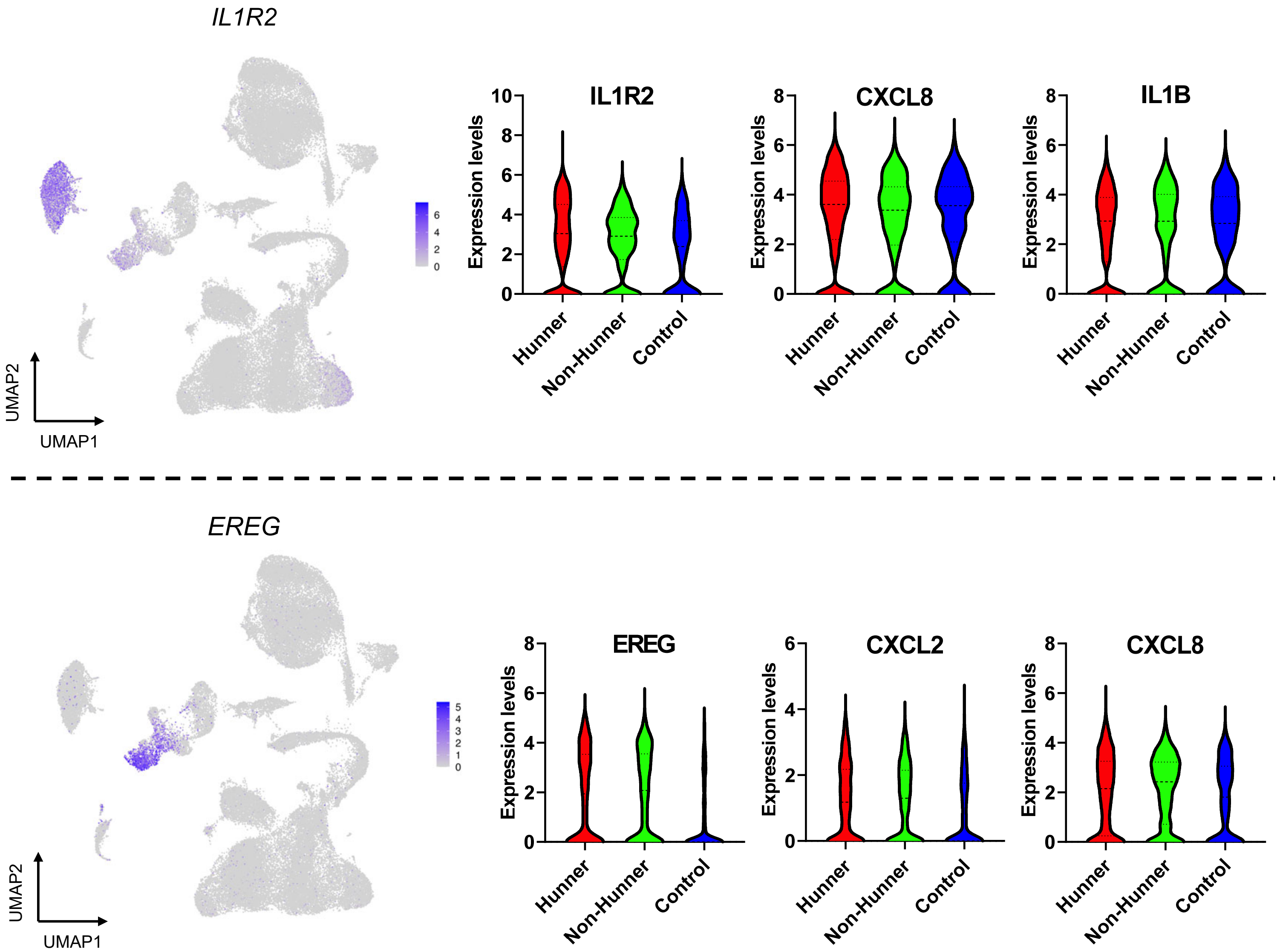

Figure S3. Characteristics of neutrophils and EREG⁺ macrophages.
Representative UMAP plots showing the expression of selected marker genes (*IL1R2* for neutrophils and *EREG* for EREG⁺ macrophages), highlighting distinct molecular features of each cluster. Violin plots depict the differential expression of genes highly expressed in neutrophils (*IL1R2*, *CXCL8*, and *IL1B*) and in EREG⁺ macrophages (*EREG*, *CXCL2*, and *CXCL8*), with comparative expression profiles illustrated across Hunner lesion, non-Hunner lesion, and non-cystitis control tissues.

A) Hunner vs Control

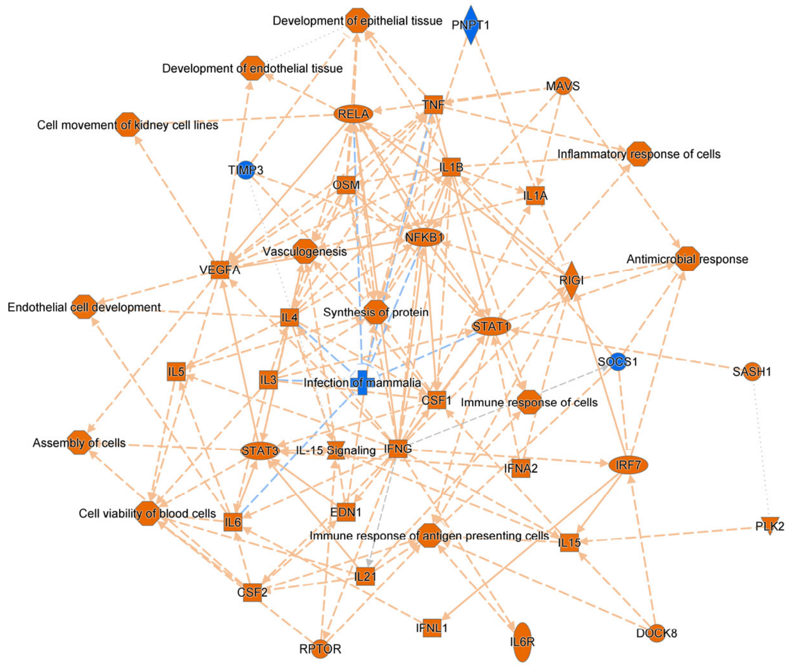

B) Hunner vs Non-Hunner

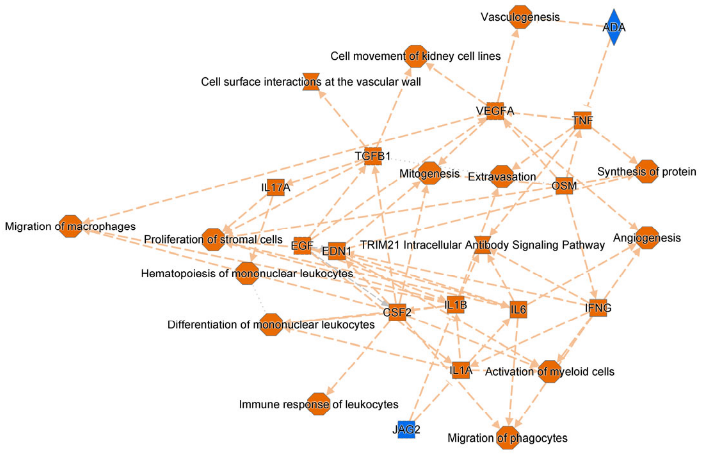

C) Non-Hunner vs Control

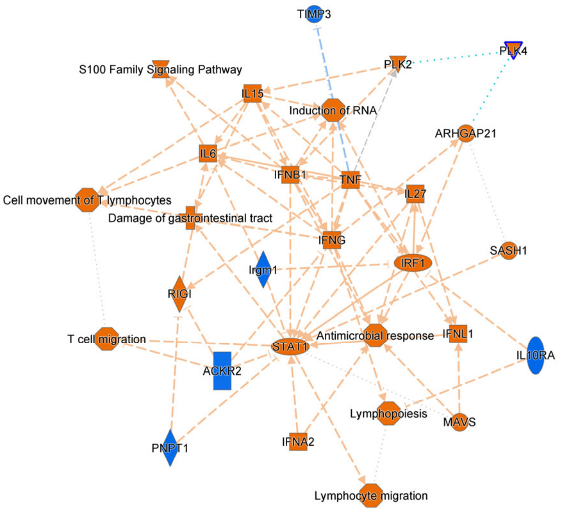

Figure S4. Pathway activation and prediction analysis in immune cells.
Ingenuity Pathway Analysis demonstrating differences in activated signaling pathways in immune cells among Hunner lesion, non-Hunner lesion, and non-cystitis control tissues.

A) Comparison between Hunner lesion and non-cystitis control tissues.
B) Comparison between Hunner lesion and non-Hunner lesion tissues.
C) Comparison between non-Hunner lesion and non-cystitis control tissues.

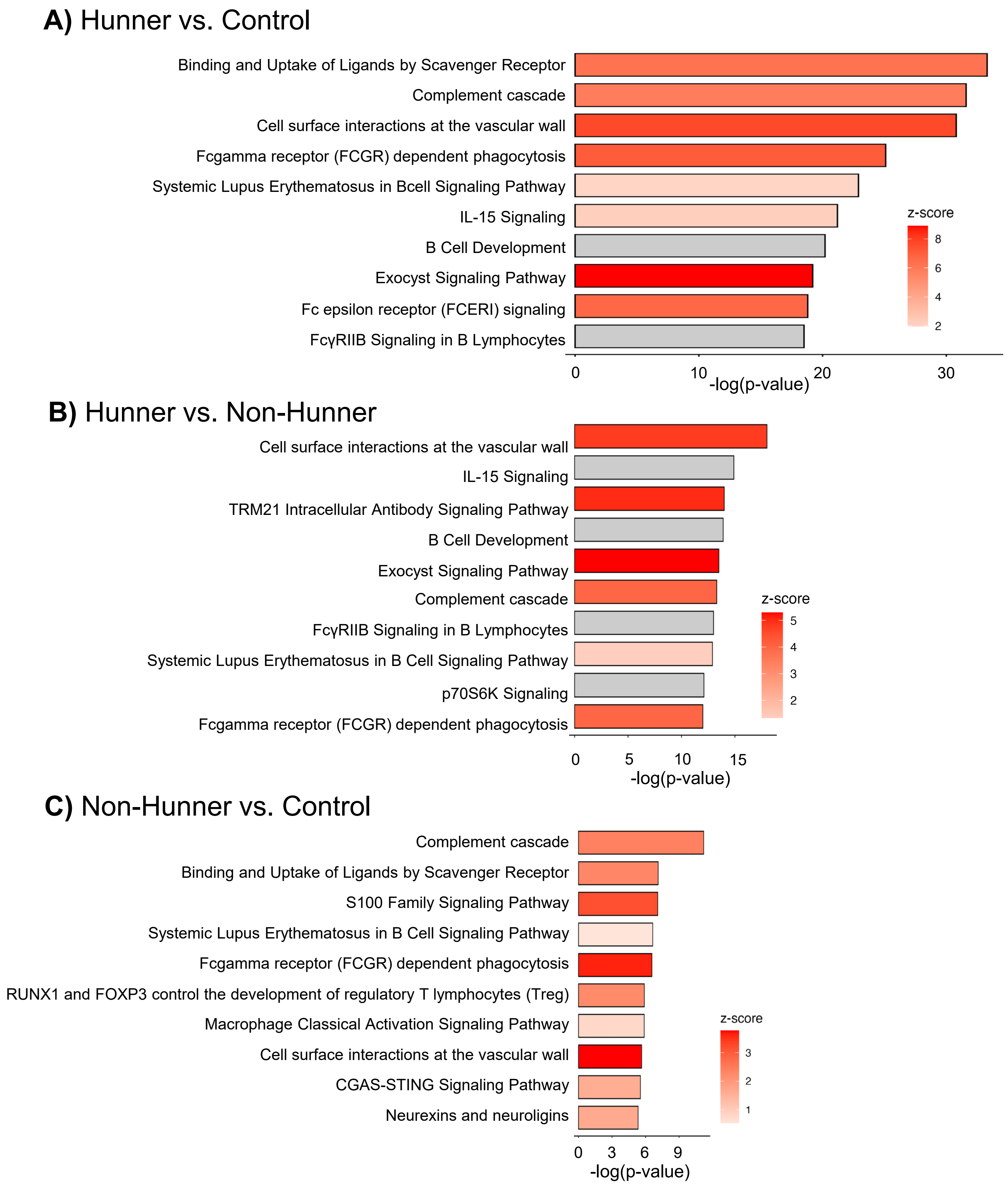

Figure S5. Canonical pathway signatures in Hunner lesions, non-Hunner lesion, and non-cystitis controls

A) Ingenuity Pathway Analysis (IPA) canonical pathways enriched in Hunner lesions compared with non-cystitis controls. Bar length indicates $-\log_{10}$(p-value), and bar color reflects activation z-score.

B) Canonical pathway differences between Hunner lesions and non-Hunner lesion, showing distinct immune- and stromal-related signaling patterns.

C) IPA pathway enrichment in non-Hunner lesion compared with non-cystitis controls. Color scales represent predicted activation (red) or inhibition (blue).

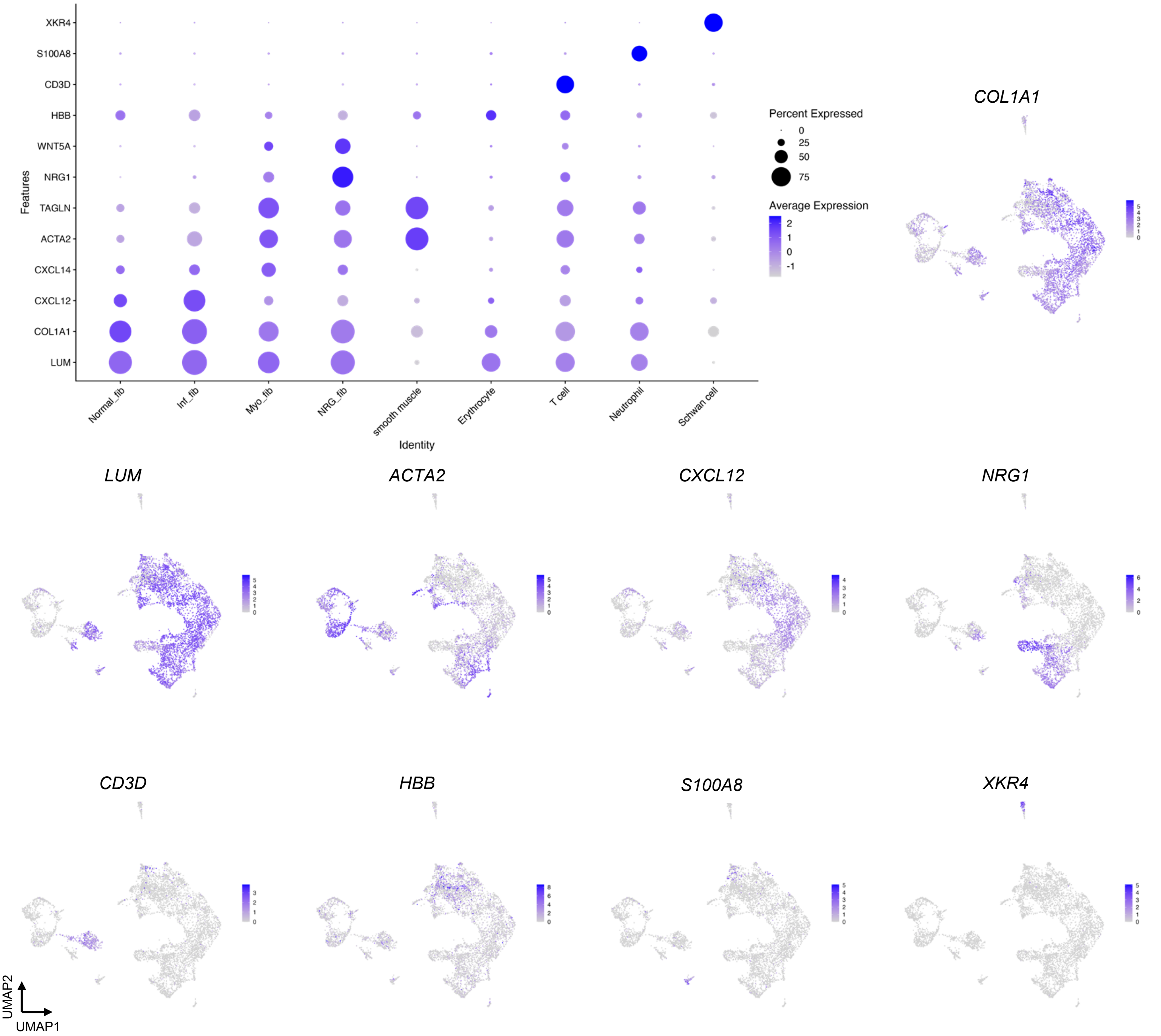

Figure S6. Fibroblast and stromal cell subclassification by canonical marker genes.
Dot plot showing representative marker gene expression across annotated fibroblast and stromal cell clusters. Dot size indicates the proportion of expressing cells, and color intensity indicates scaled average expression. Normal fibroblasts were identified by *LUM* and *COL1A1*, inflammatory fibroblasts by *CXCL12* and *CXCL14*, myofibroblasts by *ACTA2* and *TAGLN*, and NRG fibroblasts by *NRG1* and *WNT5A*. Smooth muscle cells showed high *ACTA2* and *TAGLN* expression with relatively low *LUM* and *COL1A1* expression. Erythrocytes, T cells, neutrophils, and Schwann cells were identified by *HBB*, *CD3D*, *S100A8*, and *XKR4*, respectively.

	GO terms	p value
Normal_Fib	Cytoplasmic translation	9.39E-52
	Purine ribonucleoside triphosphate biosynthetic process	1.06E-23
	Purine nucleoside triphosphate biosynthetic process	1.61E-23
Inf_FIb	Positive regulation of cell projection organization	1.04E-12
	Cell-substrate adhesion	7.77E-12
	Regulation of neuron projection development	2.10E-11
Myo_Fib	Cytoplasmic translation	1.22E-90
	Ribosome biogenesis	7.31E-27
	Ribosomal small subunit biogenesis	1.45E-20
NRG_Fib	Proteasome-mediated ubiquitin-dependent protein catabolic process	2.72E-20
	Nucleocytoplasmic transport	5.92E-18
	Nuclear transport	5.92E-18

Figure S7. Gene Ontology (GO) enrichment analysis of stromal components based on fibroblast clusters.
Representative GO terms significantly enriched in each cluster are shown with corresponding P values.

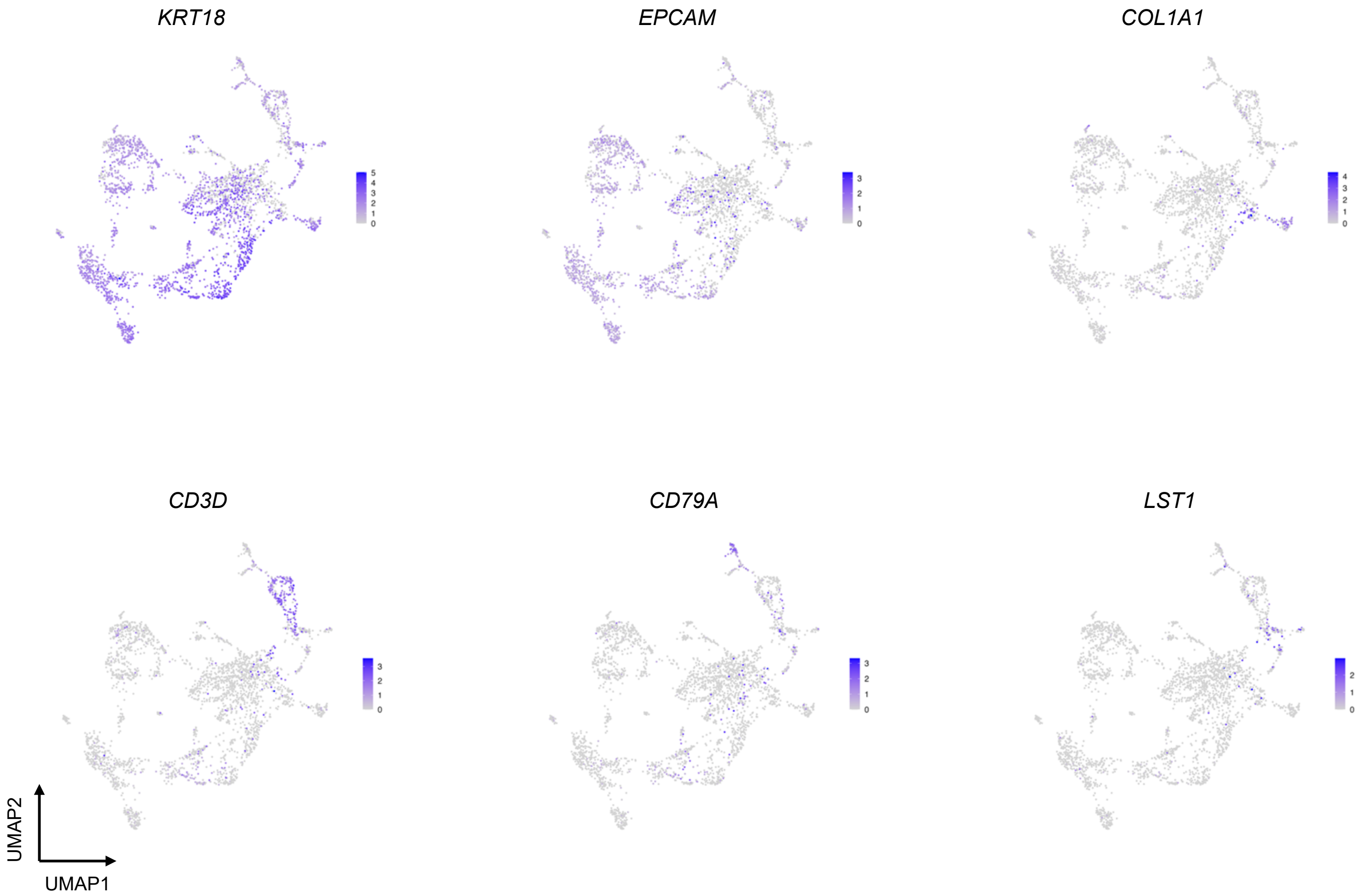

Figure S8. Representative feature plots of major cell lineage marker genes.
Feature plots showing the expression of representative marker genes: *KRT18* and *EPCAM* for epithelial cells, *COL1A1* for fibroblasts, *CD3D* for T cells, *CD79A* for B cells, and *LST1* for myeloid cells.

	GO terms	p value
Basal	Cytoplasmic translation	2.25E-33
	Ribosome biogenesis	2.51E-16
	rRNA processing	3.45E-13
Intermediate	Small GTPase mediated signal transduction	9.65E-10
	Cell-cell junction organization	3.77E-09
	Protein localization to nucleus	4.00E-09
Umbrella	Retina homeostasis	1.95E-05
Inf_Umb	Cellular respiration	1.18E-17
	Electron transport chain	1.22E-17
	mitochondrial respiratory chain complex assembly	4.07E-17

Figure S9. Gene Ontology (GO) enrichment analysis of epithelial cell clusters. Representative GO terms significantly enriched in each cluster are shown with corresponding P values.

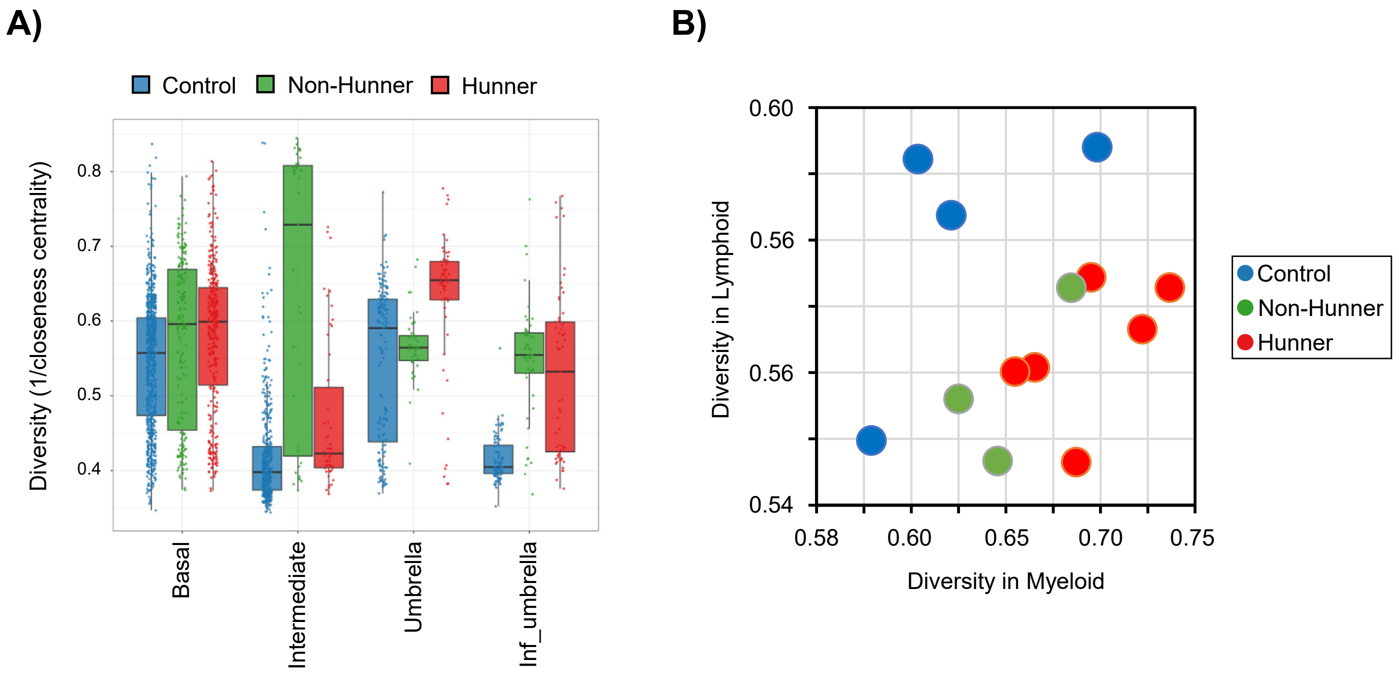

Figure S10. Closeness centrality analysis for elucidating bladder microenvironment diversity.

A) Boxplots of 1/closeness centrality in each subtype of epithelial cell among Normal, Non-Hunner, Hunner lesions. Data are presented as median (IQR).

B) Scatter plots of diversity between two cell types (lymphoid cells vs. myeloid cells). The Pearson correlation coefficients.

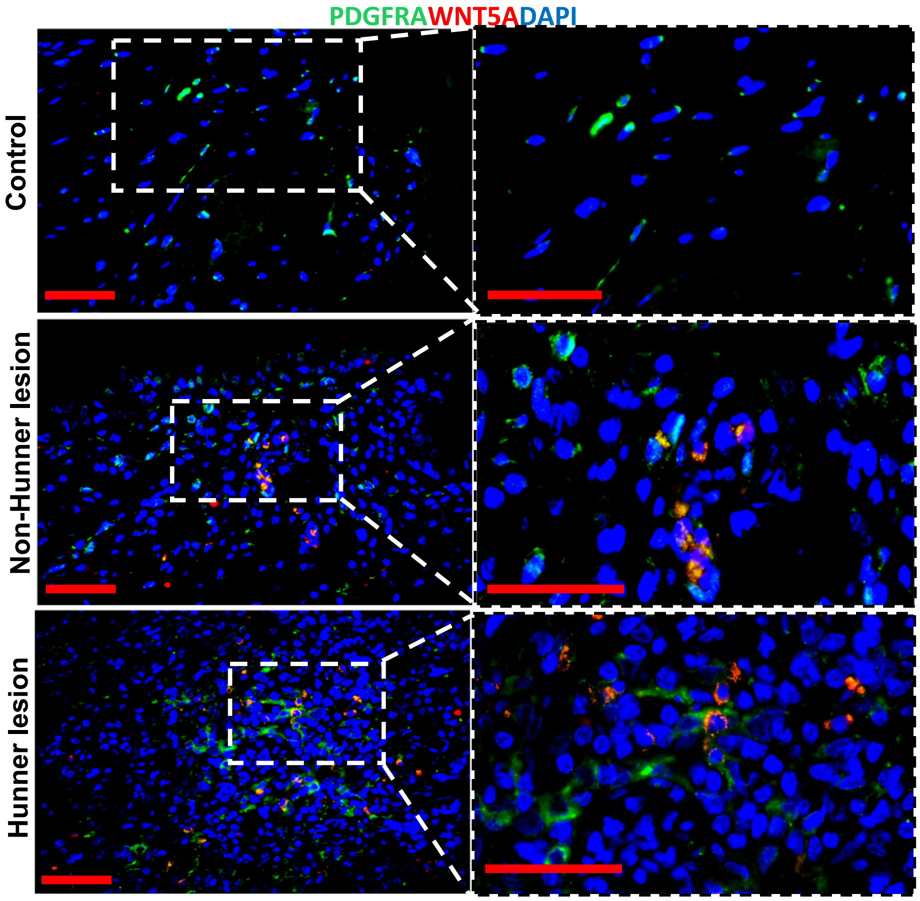

Figure S11. Immunofluorescence staining of WNT5A and PDGFRA in bladder tissue demonstrating co-localization in Hunner-lesion and Non-Hunner lesion fibroblasts. Representative immunofluorescence images showing WNT5A and PDGFRA expression in bladder tissue, with co-localization observed in fibroblasts within Hunner and Non-Hunner lesions. Nuclei were counterstained with 4′,6-diamidino-2-phenylindole (DAPI). Scale bars represent 50 μm.

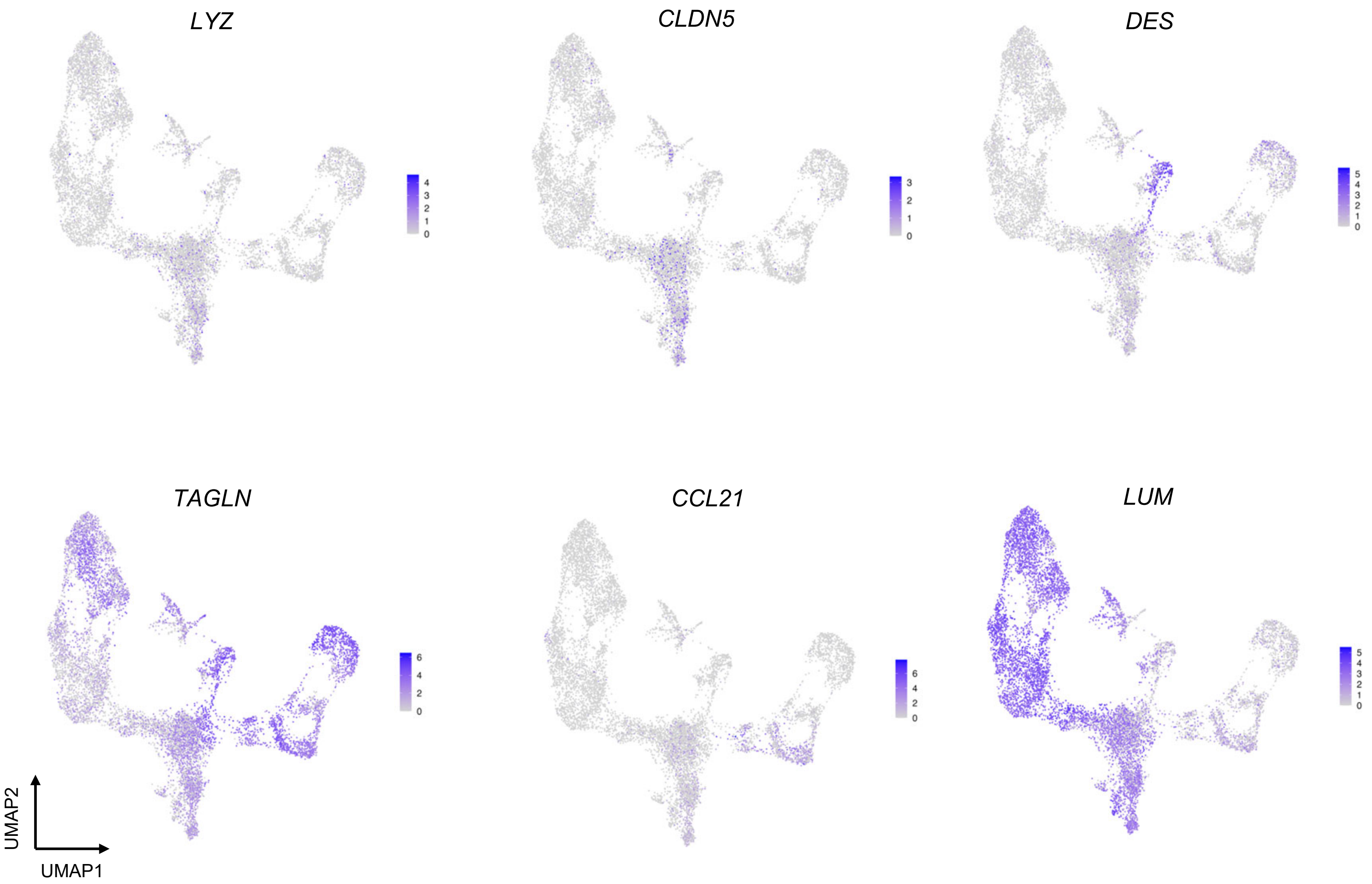

Figure S12. Representative UMAP plots showing the expression patterns of selected genes. UMAP plots illustrating the expression of representative marker genes: *LYZ* for myeloid cells, *CLDN5* for endothelial cells, *DES* and *TAGLN* for smooth muscle, and *CCL21* for lymphatic endothelial cells. LUM for stromal marker.

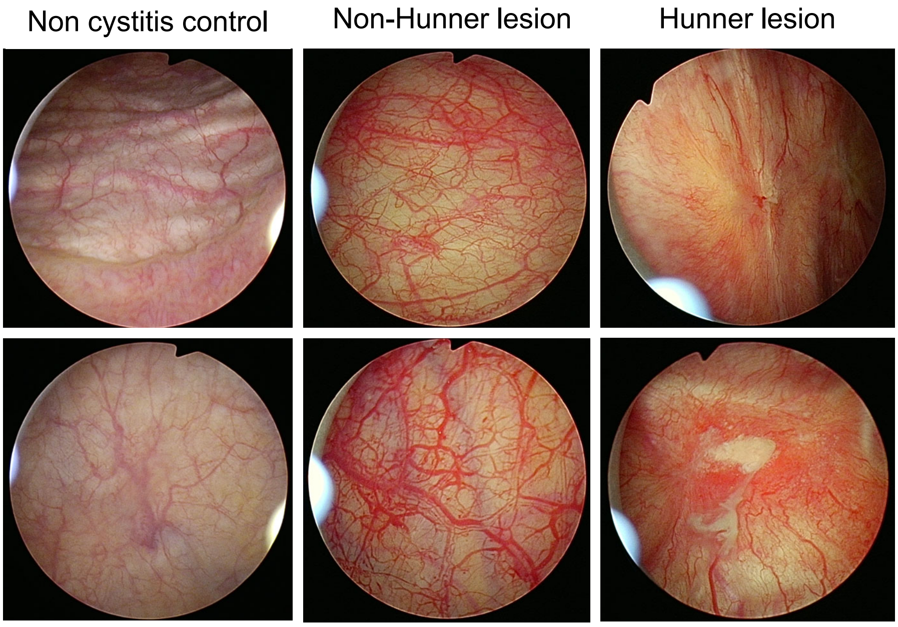

Figure S13. Cystoscopic findings in non-cystitis controls, Non-Hunner lesions, and Hunner lesions.
Representative cystoscopic images demonstrate distinct mucosal appearances across disease categories. In non-Hunner lesions, diffuse erythematous changes are observed throughout the bladder mucosa. In contrast, Hunner lesions are characterized by typical erosive lesions. From a distant view, these lesions appear as areas of erythematous mucosa. The lesion sites lack normal capillary architecture and instead show dense, tangled, mesh-like proliferation of abnormal vessels.
